# Macrolide resistance determinants and their associations in streptococci from selected livestock and wildlife species from Catalonia, Northeast Spain

**DOI:** 10.1128/spectrum.02567-25

**Published:** 2026-03-16

**Authors:** Guillem López de Egea, Aida González-Díaz, Virginia Aragón, Oscar Cabezón, Gérard Guédon, Dàmaris Berbel, Irene Cadenas-Jiménez, Johan Espunyes, Marta Planellas, M. Ángeles Domínguez, Nathalie Leblond-Bourget, Carmen Ardanuy

**Affiliations:** 1 Hospital Universitari de Bellvitge-IDIBELL-UB 16383, L'Hospitalet de Llobregat, Spain; 2 CIBER de Enfermedades Respiratorias-ISCIII 38176, Madrid, Spain; 3 Department of Pathology and Experimental Therapeutics, School of Medicine and Health Sciences, University of Barcelona 16724, Barcelona, Spain; 4 IRTA, Animal Health, Unitat mixta d’investigació IRTA-UAB en Sanitat Animal, Centre de Recerca en Sanitat Animal (CReSA), and OIE Collaborating Centre for the Research and Control of Emerging and Re-emerging Swine Diseases in Europe (IRTA-CReSA), Campus de la Universitat Autònoma de Barcelona (UAB) 571763 , Bellaterra, Spain; 5 Wildlife Conservation Medicine research group (WildCoM), Departament de Medicina i Cirurgia Animals, Universitat Autònoma de Barcelona, Bellaterra, Spain; 6 Université de Lorraine, INRAE, DynAMic 137665, Nancy, France; 7 Departament de Medi Ambient i Sostenibilitat, Ministeri de Medi Ambient, Agricultura i Ramaderia, Govern d’Andorra (Andorra), Andorra la Vella, Andorra; 8 Hospital Clínic Veterinari UAB, Campus UAB 577163 , Bellaterra, Spain; 9 Research Network for Infectious Diseases (CIBERINFEC), ISCIII 637284 , Madrid, Spain; Meijo University, Nagoya, Japan

**Keywords:** macrolide resistance, animal streptococci, mobile genetic elements

## Abstract

The increasing macrolide resistance in *Streptococcus* spp. causing human and animal infections in the last decades is a concern for global health. The objectives of this study were to analyze the macrolide resistance rates of *Streptococcus* spp. from animals and their resistance determinants. We conducted a retrospective study of an animal *Streptococcus* collection (307 isolates) from farm, wild animals, and pets in Catalonia, Northeast Spain. Identification was done by MALDI-TOF, and antimicrobial susceptibility to erythromycin and clindamycin was assessed by disk diffusion (EUCAST). Resistant strains were further tested for susceptibility to other antimicrobial agents using disk diffusion and microdilution methods. Selected isolates (*n* = 50) were subjected to whole-genome sequencing (WGS). Mobile genetic elements (MGEs), such as integrative and conjugative elements (ICEs) and integrative and mobilizable elements (IMEs), were identified using ICEscreen. Streptococcal strains were mainly isolated from domestic swine (50.5%) and wild boars (19.2%), with *S. suis* (54.4%) and *S. hyovaginalis* (14%) the predominant species. The macrolide resistance phenotypes found were MLSB (*n* = 145), M (*n* = 6), and L (*n* = 30). Macrolide (84.5%) and lincosamide (94.8%) resistance rates from swine strains were higher than those from other animals (13.2% and 18.4%, respectively, *P* < 0.001). The predominant resistant genes found were *erm*(B) (*n* = 38), *tet*(O) (*n* = 28), *vga*(F) (*n* = 20), and *lnu*(B)-*lsa*(E) (*n* = 10), and were mostly associated with ICEs or defective ICEs (dICEs) belonging to the Tn*5252* family. Animal streptococci presented high macrolide resistance rates, especially concerning swine strains, associated with a variety of resistance determinants. MGEs were the main carriers of resistance determinants and contributors to its spread.

**IMPORTANCE:**

This study adds evidence about the prevalence of macrolide and lincosamide resistance genes among streptococci from livestock, companion animals, and wildlife. It shows that streptococci from farm animals present the highest resistance rates and the presence of resistance in wild animals, like wild boars. From a One Health perspective, this study shows that resistance determinants could be shared between animal and human streptococci. It claims the need for surveillance of antibiotic resistance of human and animal streptococci in order to mitigate the emergence of resistance.

## INTRODUCTION

The *Streptococcus* genus comprises many species that are able to colonize as commensal microorganisms and also to cause infectious diseases in humans and animals (1). The most important human pathogenic species are *S. pneumoniae*, *S. pyogenes*, *S. agalactiae,* and *S. dysgalactiae* subsp. *equisimilis* (2), while the most relevant in animals depend on the animal species and include *S. suis* (swine) (3), *S. uberis* (bovines) (4), and *S. canis* (dogs and cats) (5).

In both humans and animals, streptococcal infections are treated with β-lactams as the first therapeutic option, with macrolides and lincosamides as alternative treatments (2, 6). But the increasing macrolide resistance in *Streptococcus* spp. causing human and animal infections in the last decades is making these antibiotics unsuitable for these infections, leading to a concerning problem of global health (7). A recent update of the bacterial priority pathogens list from the World Health Organization (WHO; 2024) included macrolide-resistant group A Streptococci and *Streptococcus pneumoniae* as medium group threats, because of their increase in mortality, incidence, 10-year trend of resistance, and health burden rates (www.who.int).

Macrolide resistance is mainly mediated in streptococci by two mechanisms. First, target site modification is mediated by methylases (encoded by *erm* genes), resulting in the MLSB phenotype (resistance to macrolides, lincosamides, and streptogramin B). This phenotype can be either constitutive or inducible. Second, efflux pumps encoded by *mef* genes, which result in the M phenotype (resistance to 14- and 15-membered ring macrolides) (7).

These increasing trends in antimicrobial resistance rates highlight the urgent need for surveillance within a One Health framework, as animal-associated streptococcal species could act as zoonotic pathogens and reservoirs of resistance determinants that can be transferred to human streptococcal pathogens, and vice versa (8). In this context, mobile genetic elements (MGEs) play an important role by leading horizontal gene dissemination of resistance among streptococcal species (8). Focusing on MGEs, integrative and conjugative elements (ICEs) and integrative and mobilizable elements (IMEs) are well-known carriers of resistance determinants (9, 10). The main difference between these two types of elements lies in their transmission mechanism: ICEs encode the complete machinery for self-transmission by conjugation, whereas IMEs cannot transfer autonomously and subvert the conjugation machinery of co-resident conjugative plasmids or ICEs for their transfer (9).

This study aimed to analyze the macrolide resistance rates in *Streptococcus* spp. of animal origin and to describe the presence of antimicrobial resistance determinants and MGEs carrying resistance, as well as their putative relationship with those found in human streptococci.

## MATERIALS AND METHODS

### Setting, bacterial isolates, and antibiotic susceptibility testing

We conducted a retrospective study of streptococcal species isolated from animals (carriers and infected) from a historical collection of Animal Health Research Center (CReSA-IRTA) and a pet collection from Universitat Autònoma de Barcelona (UAB) from January 2003 to December 2020. Animals from CReSA-IRTA collection included domestic swine (*Sus scrofa domesticus*; *n* = 155), wild boars (*Sus scrofa*; *n* = 59), Pyrenean chamois (*Rupicapra pyrenaica*; *n* = 44), cows (*Bos taurus*; *n* = 17), sheep (*Ovis aries*; *n* = 17), rabbits (*Oryctolagus cuniculus; n* = 2), dolphins (*Tursiops truncatus*; *n* = 2), sparrow (*Passer domesticus*; *n* = 1), and griffon vulture (*Gyps fulvus*; *n* = 1). Animals from the WildCoM-UAB collection were dogs (*Canis lupus familiaris*; *n* = 7) and cats (*Felis catus*; *n* = 2).

Isolates were identified by MALDI-TOF (Bruker Daltonics, Bremen, Germany). Antimicrobial susceptibility was studied using disk diffusion and microdilution methods following EUCAST 2025 recommendations and criteria (www.eucast.org). Clinical breakpoints for *Streptococcus* groups A, B, C, and G were applied to all streptococcal species. All strains were tested using disk diffusion for erythromycin and clindamycin susceptibility. Strains showing macrolide or lincosamide resistance were further tested against other antibiotics using disk diffusion and microdilution, as shown in the study flow chart (Fig. S1). Other antibiotics tested were penicillin, cefotaxime, linezolid, tetracycline, chloramphenicol, levofloxacin, vancomycin, and trimethoprim-sulfamethoxazole. The macrolides-lincosamides resistance phenotype was assessed by the D-test (11).

### Whole-genome sequencing analysis

The molecular characterization of selected strains was performed using whole-genome sequencing (WGS). A subset of 50 strains was selected to perform WGS (selection criteria specified in Fig. S1). The DNA was extracted using the QIAamp DNA Mini Kit (Qiagen, Hilden, Germany) and quantified with Qubit (Thermo Fisher Scientific, Waltham, MA, USA). Library preparation was done with Nextera XT and sequenced on an Illumina MiSeq Platform (Illumina, San Diego, CA, USA). Long-read sequencing was performed using the Ligation Sequencing Kit (SQK-LSK109) and followed by sequencing on FLO-MIN106D flow cells (R9) and using the Fast model for basecalling (Oxford Nanopore Technologies, Oxford, UK). Bioinformatic analysis was conducted using the Bactopia pipeline (12), which includes reads quality control (fastQC), assembly (Shovill), annotation (Prokka), sequence typing (mlst), and antibiotic acquired resistance scan (AMRFinder+). Hybrid assembly was conducted with Unicycler. Conjugative or mobilizable genetic elements annotation was conducted with ICEscreen (13). In this study, the two most abundantly detected ICE families by ICEscreen were Tn*916* and Tn*5252*. These elements notably possess distinct coupling proteins: those of the Tn*916* family are related to FtsK/spoIIIE proteins (pfam01580), whereas those of the Tn*5252* family are related to VirD4 proteins (pfam02534). These families also differ by their relaxase type, with Tn*916* being associated with reptrans-like relaxases (pfam02486) and Tn*5252* with MOBP-type relaxases (pfam03432). MGEs associated with antibiotic resistance were screened with Geneious R9 (Biomatters, Auckland, New Zealand). For the phylogenetic tree reconstruction of all genomes from all *Streptococcus* species, a pangenome analysis was conducted using Roary, with a minimum BLASTp identity threshold of 80% and without splitting paralogs. The 223 genes present in all genomes were used to reconstruct the tree with IQ-tree, using the GTR+F+I+G4 model and 1,000 UFBoot replicates, and the tree was rooted at the midpoint. The phylogenetic tree of *S. suis* was reconstructed using the alignment obtained by Snippy and IQ-tree with the same GTR+F+I+G4 model and 1,000 UFBoot replicates, also rooted at the midpoint. *S. suis* BM407 (FM252032.1) was used as the reference strain. Both phylogenetic analyses and data were visualized with iTol (itol.embl.de/). Integrases, relaxases, and VirB4 proteins from Tn*5252*-family ICEs or defective ICEs (dICEs) were classified into previously described clades by Huang et al. (8). Protein sequences were aligned using Clustal Omega, and phylogenetic trees were constructed using the neighbor-joining method. Sequence comparisons of Tn*5252*-family ICEs and prophages were visualized using the Easyfig program. Extended bioinformatic methodology is summarized in Table S1. Reads were deposited at the European Nucleotide Archive (PRJEB95797, Table S2).

### Statistical analysis

Chi-square test was used to assess differences in macrolide and lincosamide resistance rates between strains isolated from swine and those from other animal species.

## RESULTS

### Presence of different streptococcal species between farm, wildlife, and companion animals

A total of 307 streptococcal strains isolated from livestock, companion animals, and wildlife species were selected from IRTA-CReSA and WildCoM-UAB bacterial collections (Fig. S1). The streptococcal strains of these collections belonged to farm animals (*n* = 189), wild animals (*n* = 108), and companion animals (*n* = 10). Of these, 155 were isolated from domestic swine (*Sus scrofa domesticus*; 50.5%), 59 from wild boars (*Sus scrofa*; 19.2%), 44 from Pyrenean chamois (*Rupicapra pyrenaica*; 14.3%), 17 from cows (*Bos taurus*; 5.5%), 17 from sheep (*Ovis aries*; 5.5%), 7 from dogs (*Canis lupus familiaris*; 2.3%), 2 from cats (*Felis catus*; 0.7%), 2 from rabbits (*Oryctolagus cuniculus*; 0.7%), 2 from dolphins (*Tursiops truncatus*; 0.7%), 1 from a sparrow (*Passer domesticus*; 0.3%), and 1 from a griffon vulture (*Gyps fulvus*; 0.3%).

The *Streptococcus* species found were *S. suis* (54.4%), *S. hyovaginalis* (14.0%), *S. lutetiensis* (5.5%), *Streptococcus* spp. (3.9%), *S. uberis* (3.3%), *S. entericus* (3.3%), *S. pluranimalium* (2.0%), *S. dysgalactiae* subsp. *dysgalactiae* (2.0%), *S. gallolyticus* (1.6%), *S. pasteuri* (1.6%), and others (8.4%). *S. suis, S. hyovaginalis,* and *S. pluranimalium* were mainly found in swine and wild boars, *S. lutetiensis, S. pasteuri,* and *S. gallolyticus* were mainly found in Pyrenean chamois, *S. uberis* and *S. dysgalactiae* subsp. *dysgalactiae* were exclusively found in cows, and *S. entericus* was exclusively found in sheep (Table S3).

### Higher macrolide resistance rates were associated with farm and companion animals

**Fig 1:**
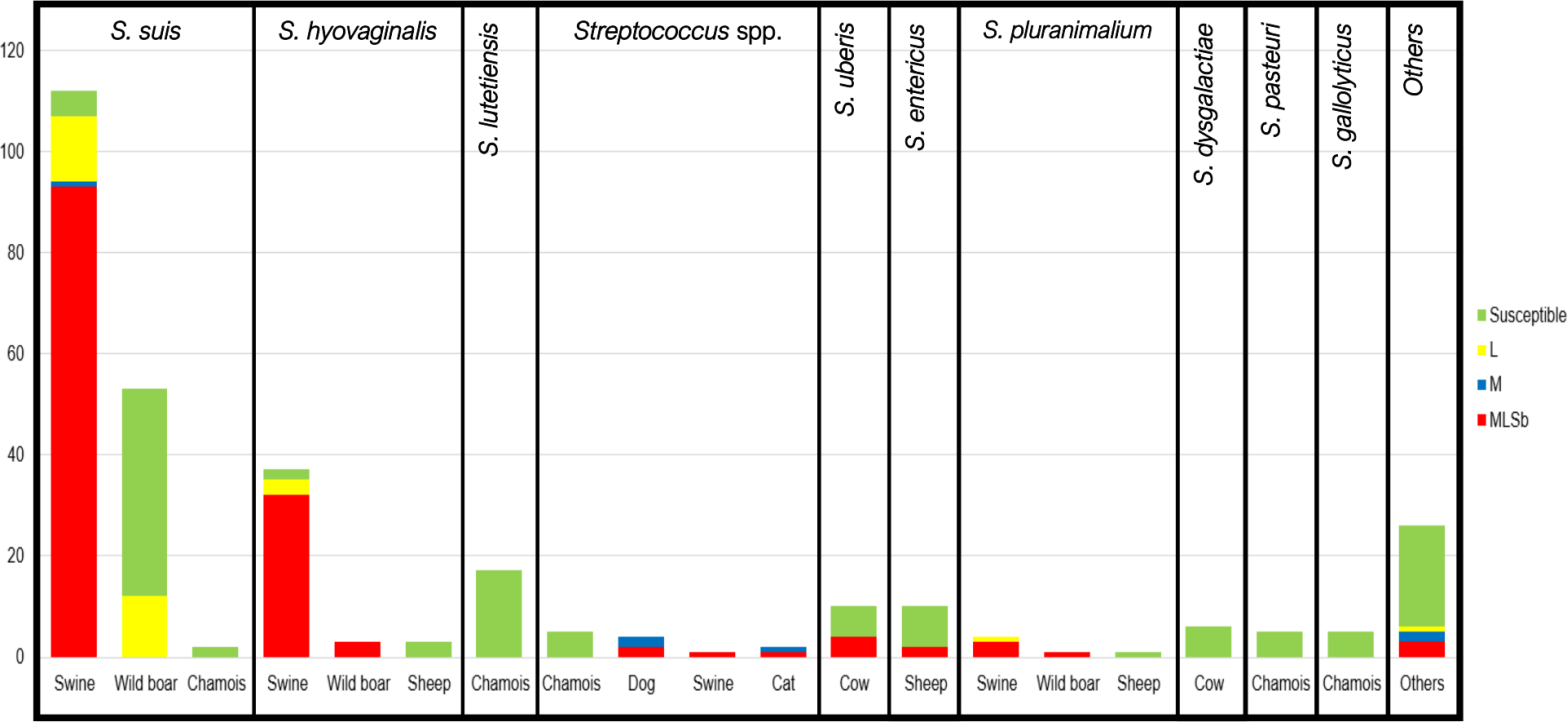
Macrolide-resistant phenotypes classified by *Streptococcus* species and animal origin. Number of strains presenting MLSB phenotype (red), M phenotype (blue), L phenotype (yellow), or susceptible phenotype (green) clustered in different streptococcal species (*S. suis, S. hyovaginalis, S. lutetiensis, Streptococcus* spp.*, S. uberis, S. entericus, S. pluranimalium, S. dysgalactiae, S. pasteuri, S. gallolyticus,* and others) by animal origin (swine, wild boar, chamois, sheep, griffon vulture, dog, cat, cow, and others).

Overall, resistance rates were 49.2% for macrolides and 57.0% for lincosamides. The macrolide-lincosamide resistance phenotypes found were MLSB (*n* = 145), M (*n* = 6), and L (resistance to lincosamides) (*n* = 30). Focusing on the most frequent streptococcal species, these phenotypes were represented by animals in Fig. 1. All *S. lutetiensis*, *S. dysgalactiae* subsp. *dysgalactiae*, *S. pasteuri,* and *S. gallolyticus* isolates presented a susceptible phenotype and were isolated from wildlife. Other frequent streptococci (*S. suis*, *S. hyovaginalis, S. uberis, S. entericus, S. pluranimalium,* and *Streptococcus* spp.) showed a variable rate of resistance phenotypes, with *S. suis* (95.5%)*, S. hyovaginalis* (94.6%), and *S. pluranimalium* (100%) from swine (farm animal) presenting the highest resistance rates (Fig. 1). Macrolide (84.5%) and lincosamide (94.8%) resistance rates from swine strains (*n* = 155) were significantly higher (macrolides: *X^2^* = 156.35, *df* = 1, *P* < 0.001; lincosamides: *X^2^* = 182.85, *df* = 1, *P* < 0.001) than those collected from other animals (*n* = 152) (13.2% and 18.4%, respectively). Among streptococci from wild animals, macrolide resistance was mainly detected in those collected from wild boars (27.1%). No resistance phenotype was found in streptococcal strains from Pyrenean chamois and rabbits. Streptococci isolated from companion animals (dogs and cats) showed 77% (7/9) of resistance to macrolides and 44% (4/9) of resistance to lincosamides.

Strains presenting macrolide or lincosamide resistance (*n* = 181) were frequently resistant to other antibiotic classes: tetracycline (152/181, 83.9%), co-trimoxazole (83/181, 45.8%), quinolones (43/181, 23.7%), penicillin (21/181, 11.6%), and chloramphenicol (12/181, 6.6%) (Table S4). Of these strains, 163 (90.0%) were resistant to more than one antibiotic class. This co-resistance pattern was mainly observed in domestic swine (146/148, 98.6%). Additionally, other streptococci isolated from wildlife and companion animals presented lower co-resistance rates (17/33, 51.5%).

### Genetic relationship between streptococci from farm, wildlife, and companion animals

**Fig 2:**
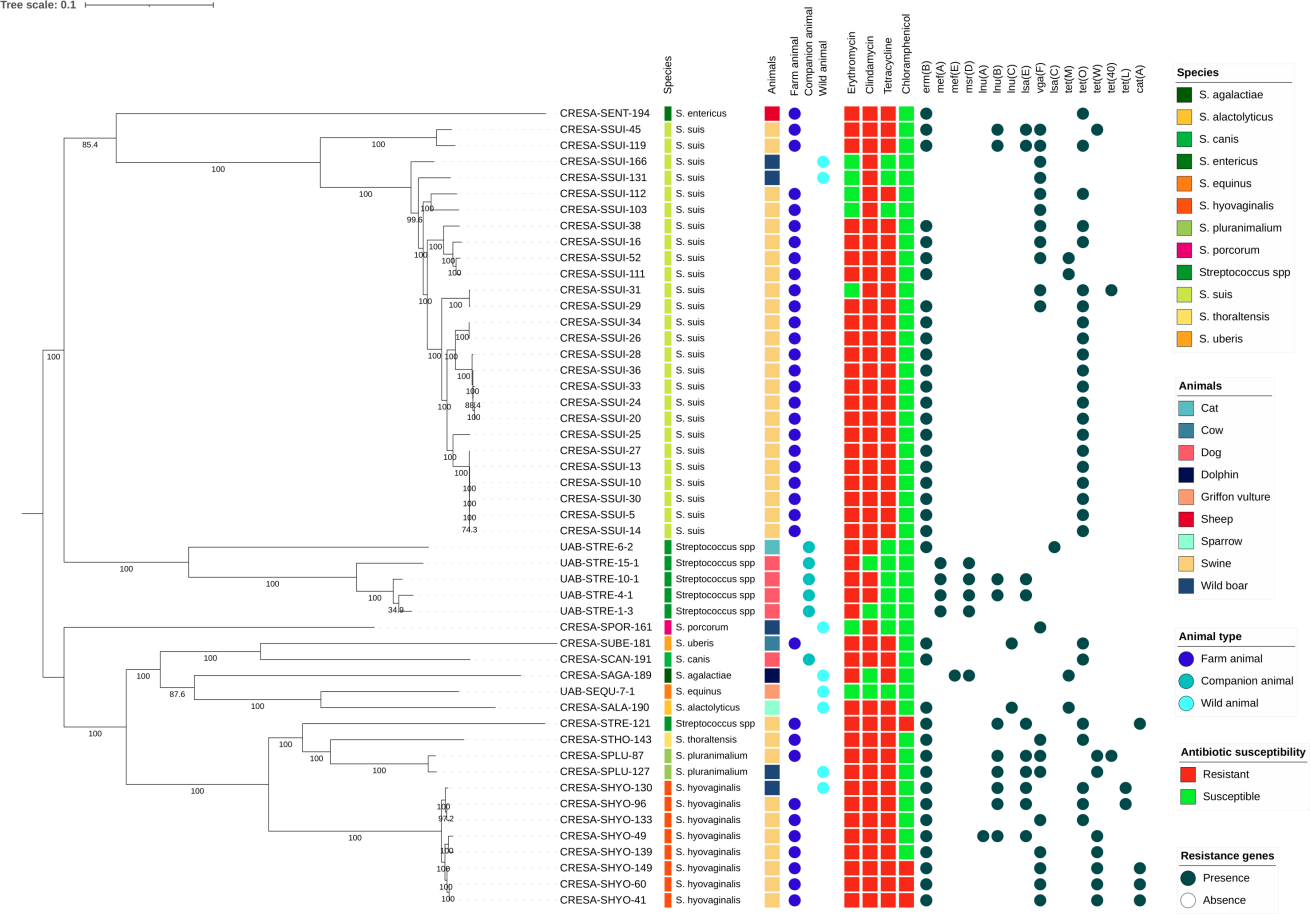
Phylogenetic tree and presence/absence matrix depicting acquired antibiotic resistance genes associated with resistance of *Streptococcus* spp. from animals. The branches of the phylogenetic tree include the strain identification. The bootstrap values (percentages of 1,000 replicates) are depicted at the branching points. The first part includes *Streptococcus* species (colored rectangles in the first column); data of the animal from which streptococci were isolated (second column squares); and animal type: farm, companion, and wild animals (colored circles in the third, fourth, and fifth columns). The second part includes a matrix of columns with squares representing antibiotic susceptibility testing results: red (resistant) and green (susceptible); the antibiotic is referred to at the top. The last section shows the presence (dark blue circle) or absence (no circle) of different acquired resistance genes associated with antibiotic resistance.

A phylogenetic tree was performed with selected strains subjected to WGS (Fig. 2). A high genetic diversity, inter- and intra-species, was observed. Classifying by species, four differentiated groups were identified: *S. suis* from farm and wild animals, *Streptococcus* spp. from companion animals, *Streptococcus* spp. from wild animals, and *S. hyovaginalis*, *S. pluranimalium,* and *S. thoraltensis* from farm and wild animals. Resistance patterns among these groups were different. The more common pattern encompassing all groups was resistance to macrolides, lincosamides, and tetracycline, mostly associated with *erm*(B) and *tet*(O) genes. *S. suis* from farm and wild animals showed a high rate of this mentioned pattern; however, these isolates also showed the highest presence of L phenotype (only resistance to lincosamides), which was associated with the *vga*(F) gene. The group of *Streptococcus* spp. from companion animals presented MLSB and M phenotypes, and most strains lacked tetracycline resistance. *Streptococcus* spp. from wildlife were the most heterogeneous for resistance patterns. Finally, *S. hyovaginalis*, *S. pluranimalium,* and *S. thoraltensis* from farm and wild animals’ groups were completely resistant to macrolides, lincosamides, and tetracycline, and some strains showed resistance to chloramphenicol.

Focusing only on *S. suis*, a high genetic diversity was found (Fig. S2). A total of 18 sequence types (STs) were found, with ST1 (4/26) and ST123 (4/26) being predominant. The serotypes found were heterogenic (10 different serotypes), with serotype 2 (4/26), serotype 9 (4/26), and non-typeable strains (5/26) being more frequent. In order to assess the absence of polysaccharide capsule in non-typeable isolates, we compared the sequences of all available capsular operons of *S. suis* from Okura et al. (14) with our strain sequences. Most strains exhibited MLSB phenotype and tetracycline resistance (21/26), while some showed L phenotype (5/26) with or without tetracycline resistance. The more prevalent resistant genes found on *S. suis* were *erm*(B), *tet*(O), and *vga*(F).

Overall WGS analysis of macrolide or lincosamide-resistant selected strains showed the presence of the following resistance determinants: *erm*(B) (*n* = 38), *mef*(A)-*msr*(D) (*n* = 4), *mef*(E)-*msr*(D) (*n* = 1), *lnu*(A) (*n* = 1), *lnu*(B)-*lsa*(E) (*n* = 10), *lnu*(C) (*n* = 2), *lsa*(C) (*n* = 1), and *vga*(F) (*n* = 20) (Fig. 2). Additional resistome analysis found resistance determinants for tetracyclines and amphenicols: *tet*(M) (*n* = 4), *tet*(O) (*n* = 28), *tet*(W) (*n* = 8), *tet*(40) (*n* = 2), *tet*(L) (*n* = 2), and *cat*(A) (*n* = 4). No β-lactamase genes were detected in penicillin-resistant strains.

### Mobile genetic elements as main resistance carriers

**Fig 3:**
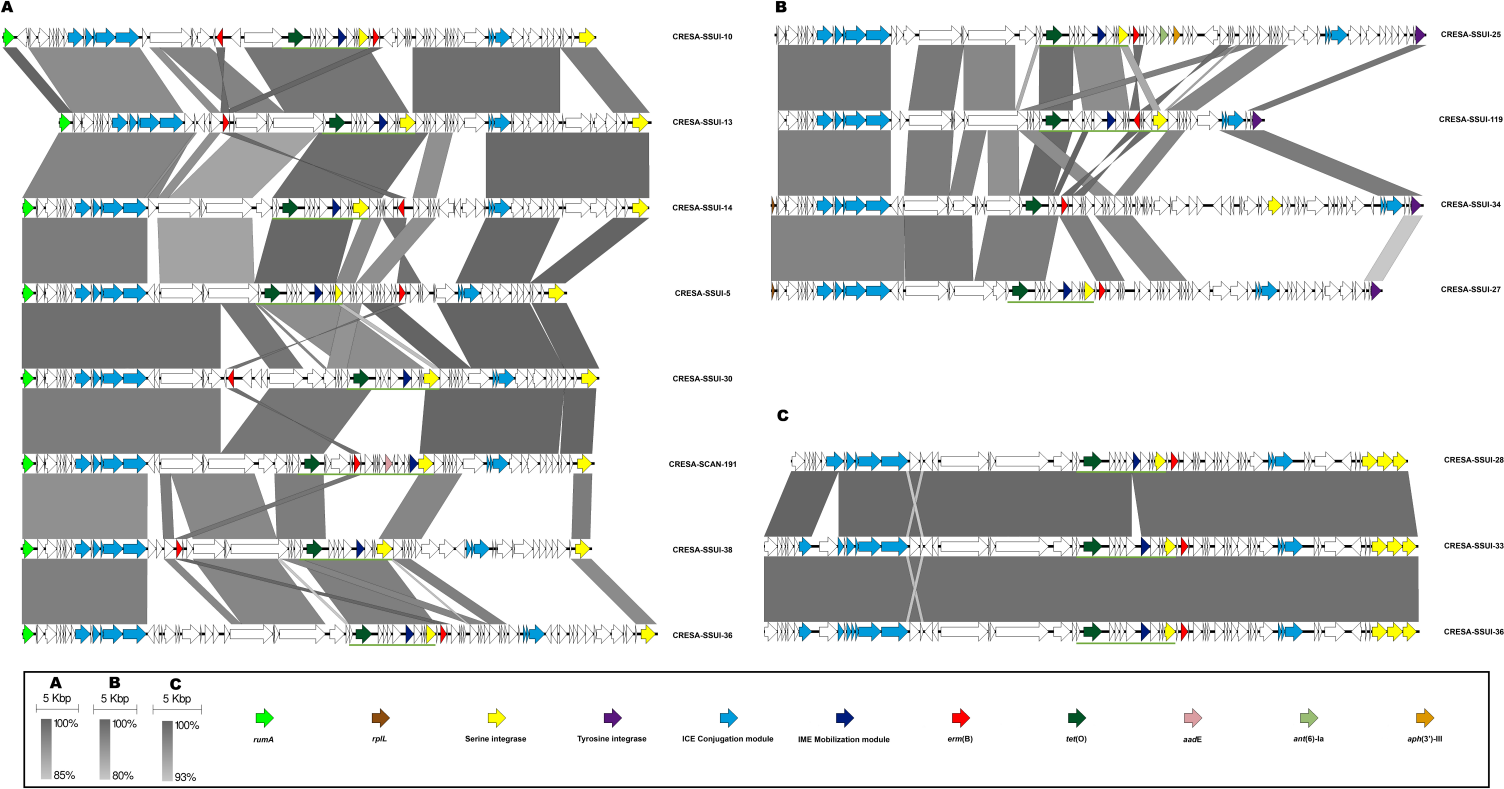
Schematic representation of Tn*5252* composite ICEs found in *Streptococcus* spp. from animals. This figure shows ICEs belonging to the Tn*5252* family carrying antibiotic resistance determinants inserted at different genomic loci: *rumA* (**A**), *rplL* (**B**), and *mutT* (**C**). Gray-to-white shaded areas connect regions based on similarity (identity from 80% to 100%). Arrows depict the genes present in each element. Colored arrows represent different genes: *rum*A (light green), *rpl*L (brown), ICE conjugation module (light blue), IME mobilization module (dark blue), serine integrase (yellow), tyrosine integrase (dark purple), *erm*(B) (red), *tet*(O) (dark green), *aad*E (pink), *ant*(6)-Ia (olive green), and *aph*(3′)-III (orange). IMEs inserted within ICEs of the Tn*5252* family are underlined in green.

All strains subjected to WGS were analyzed for the presence of both ICEs and IMEs carrying resistance determinants. The classification of ICE families was based on their conjugation modules, and IMEs were classified into superfamilies based on their relaxase, as previously described (10, 15). The predominant MGEs identified were composite elements composed of ICEs or dICEs belonging to the Tn*5252* family and carrying additional elements, such as IMEs (Table S5). Members of the Tn*5252* family of ICEs (*n* = 15) were found at different genomic loci, including *rumA* (*n* = 8), *rplL* (*n* = 4), and *mutT* (*n* = 3) (Fig. 3). The resistance genes carried by these ICEs were *erm*(B) (*n* = 15), *tet*(O) (*n* = 15), *ant*(6)-Ia (*n* = 1), *aph*(3′)-III (*n* = 1), and *aadE* (*n* = 1). The Tn*5252-*family of dICEs (*n* = 18) was also found inserted at the same genomic loci as ICEs and carried a broader range of resistance genes, including *erm*(B) (*n* = 14), *tet*(O) (*n* = 9), *tet*(W) (*n* = 8), *ant*(6)-Ia (*n* = 6), *aph*(3′)-III (*n* = 4), *cat*(A) (*n* = 4), *tet*(40) (*n* = 2), *lnu*(B)-*lsa*(E) (*n* = 2), *aadE* (*n* = 1), and *aph*(2″)-III (*n* = 1) (Table S5). Notably, dICEs belonging to the Tn*5252* family more frequently harbored more than two resistance genes (10/18) compared with ICEs of the same family (2/15). Based on the classification of integrase, relaxase, and VirB4 clades proposed by Huang et al. (8), the following clades were identified among Tn*5252* ICEs and dICEs: integrase clade III (*n* = 8), integrase clade IV (*n* = 8), integrase clade Va (*n* = 1), integrase clade Vb (*n* = 6), relaxase clade I (*n* = 8), relaxase clade II (*n* = 8), relaxase clade III (*n* = 2), relaxase clade IVa (*n* = 3), relaxase clade IVb (*n* = 2), and VirB4 clade IIIb (*n* = 26) (Fig. S3). Other ICEs identified belonged to the Tn*916* family and carried the *tet*(M) gene (*n* = 3). IMEs belonging to the MOBV superfamily were the most frequently detected IMEs; most of them were found carrying resistance genes and were inserted within ICEs or dICEs (Table S5). In addition, prophages were identified as other MGEs carrying resistance genes, including *vga*(F) (*n* = 7), *erm*(B) (*n* = 2), *ant*(6)-Ia (*n* = 2), *aph*(3′)-III (*n* = 1), and *lnu*(B)-*lsa*(E) (*n* = 1) (Fig. S4).

## DISCUSSION

This study addressed the issue of macrolide and lincosamide resistance in animal streptococci through an in-depth analysis of resistance rates, associated genes, and related MGEs. Together, these findings provide crucial information for understanding the dissemination of macrolide resistance determinants in streptococci from a One Health perspective.

First, this study shows the prevalence of macrolide and lincosamide resistance in streptococci isolated from farms, wildlife, and companion animals. Focusing on farm animals, our data showed high rates of macrolide and lincosamide resistance. These results are in line with other studies reporting high resistance rates, primarily in *S. suis,* where we found 83.9% of macrolide resistance and 94.6% of lincosamide resistance, mainly due to the presence of the *erm*(B) gene. For instance, a study in Lleida (Catalonia, Northeast Spain) detected the presence of the *erm*(B) gene in 86.4% of *S. suis* causing swine disease (16) and a European study (including Belgium, France, Germany, Spain, Hungary, Netherlands, and United Kingdom) found 65% of *S. suis* with *erm*(B) gene infecting swine (17). Regarding *S. uberis,* our results showed a high rate of macrolide and lincosamide resistance (40.0%). Similar results were reported in Maeda et al. (18), which found that 34% of *S. uberis* strains from bovine mastitis in Japan were resistant, associated with ICEs carrying *erm*(B) plus *tet*(O). In contrast, a study of Sweden detected only 1.7% of macrolide resistance in *S. uberis* strains from bovine mastitis [*erm*(B) and *mef*(A)] (19). Furthermore, we have also characterized resistance rates in other streptococcal species like *S. hyovaginalis,* where we found 80.0% and 87.5% of macrolide and lincosamide resistance in farm animals, respectively. These findings are similar to those reported in *S. hyovaginalis* causing infection in swine in Brazil (20).

On the other hand, concerning streptococci from companion animals, our findings [77.7% of macrolide resistance, 44.4% of lincosamide resistance, and detection of *erm*(B) and *mef*(A) genes] are in line with the literature. A Hungarian metagenomic study found multiple resistance genes in canine saliva and detected macrolide resistance determinants, such as *erm*(B), in *Streptococcus* spp. (21). Different macrolide resistance rates have been found in other studies from dogs and cats, being 55.4% in *S. canis* from Poland and 19.8% in *Streptococcus* spp. (*S. canis*, *S. agalactiae*...) from Japan (22, 23).

In wildlife, antibiotic resistance determinants have been studied and detected in different bacteria, e.g., the *mec*A gene in *Staphylococcus aureus* from migratory rooks, extended-spectrum β-lactamase-producing *Escherichia coli* from wild boars, and the *van*A gene in *Enterococcus faecium* from Iberian wolves, among others (24). However, to the best of our knowledge, macrolide and lincosamide resistance in streptococci of wildlife origin has not been reported yet. Our study reveals that resistance levels are low, with wild boar being the primary species that presents resistant streptococci (17/59, 28.8%). This fact could be explained by the likelihood that, despite being wild animals, wild boars often come into contact with livestock and urban areas (25). The streptococcal strains with resistance determinants found in wild boars were mainly *S. suis*. Resistance rates in these strains were lower than those observed in *S. suis* strains from domestic swine, as reported in a French study (6). In agreement, a similar pattern has been observed in vultures, where captive individuals were more likely to carry resistant strains compared to their wild counterparts (26).

From a zoonotic perspective, among the sequenced isolates, we identified *S. suis* serotype 2/ST1 (clonal complex 1), a lineage classically linked to high virulence and invasive disease in both pigs and humans (27, 28). Notably, zoonotic *S. suis* infections caused by the serotype 2/ST1 clone have also been documented in our region (29). These local reports support the zoonotic potential of the virulent serotype 2/ST1 lineage and reinforce the need for integrated One Health surveillance connecting animal reservoirs and human invasive disease.

The high resistance rates to macrolides and lincosamides shown in this work related to farm animals, as well as those described in previous studies (16–18), may be attributed to the long-term use of antibiotics to promote animal growth (a practice banned in Europe since 2006) or for metaphylactic treatments that may have selected resistant lineages. The addition of antimicrobials to animals’ farm diet enhances feed efficiency but also contributes to the increase of bacterial resistance rates, even if these antibiotics are non-medically important, such as ionophore feed additives (30). Kwon et al. (30) compared the resistome and microbiota of cattle raised in grass-fed versus grain-fed production systems and showed that cattle fed a grain diet with feed additives had higher resistance rates to medically important antibiotics, such as macrolides. Streptococcal strains from animals have zoonotic potential and, in addition, represent a reservoir of resistance determinants that could be transferred to human strains and vice versa (8). In fact, a study found the association between the use of macrolides in producing food animals and the increase of macrolide resistance in human pneumococci (31). These facts led to the scenario that macrolides are no longer a suitable alternative to β-lactam antibiotics for the treatment of streptococcal infections in animals and humans.

The macrolide, lincosamide, and tetracycline resistance determinants in our study were primarily associated with MGEs. Focusing on *S. suis*, the predominant resistance genes were *erm*(B) and *tet*(O), mainly carried by Tn*5252* composite elements, as previously reported (32). We identified *rumA*, *rplL,* and *mutT* genes as insertion sites for Tn*5252* elements. In line with this, we previously identified *rplL* as an insertion site for Tn*5252* in *S. pyogenes* (33) or both *rplL* and *rumA* in *S. pneumoniae* strains causing human infections (34). The presence of Tn*5252* insertions at shared loci suggests the potential for horizontal transmission of these elements between human and animal streptococcal species. In this context, Tn*5252* elements were the main MGEs associated with macrolide, lincosamide, and tetracycline resistance in our study, similar to findings in *S. pneumoniae* from human infections (34). This contrasts with other human streptococcal pathogens, such as *S. pyogenes* and *S. dysgalactiae* subsp. *equisimilis,* where resistance was primarily linked to Tn*1549* elements (33, 35).

The increase in macrolide resistance rates in streptococci is a concerning problem of global health. In response, the WHO included macrolide-resistant group A Streptococci and *S. pneumoniae* as medium group threats due to their increasing health burden, incidence, and associated mortality rates (www.who.int). From a One Health perspective, there is a pressing need for global surveillance of antimicrobial resistance in the predominant streptococcal species causing infections in both humans and animals. Moreover, implementing national antimicrobial stewardship programs in primary care, hospitals, and veterinary settings is essential to optimize antibiotic use and mitigate the emergence of resistance. In this context, Spain achieved a reduction of approximately 70% in antibiotic use in domestic swine between 2014 and 2020 (https://resistenciaantibioticos.es/es), following policies promoted by the “Plan Nacional frente a la Resistencia a los Antibióticos” (PRAN), in an effort to soften the rise of resistance rates.

This study has, however, some limitations. It is a retrospective study based on a specific collection of strains, mainly composed of streptococci from farm animals, particularly swine, which may have led to an overrepresentation of certain streptococcal species (especially *S. suis*) and antibiotic resistance profiles (mainly macrolides and tetracyclines). The number of isolates from some streptococcal species was low, limiting the ability to extract significant conclusions from those groups. Antibiotic use policies in animal health changed over the study period, particularly following the implementation of national antimicrobial stewardship initiatives, such as the PRAN. These policy changes may have influenced the resistance patterns observed overall in farm animals. Despite these limitations, the study’s strengths include its relatively large sample size, the variety of animal species included, and the application of WGS analysis.

In conclusion, this study highlights the high prevalence of macrolide and lincosamide resistance in streptococci from farm animals, particularly *S. suis*, mainly due to the *erm*(B) gene carried by MGEs, such as Tn*5252*. Resistance was also detected in isolates from companion animals, while it remained very low in wildlife, with the exception of wild boars, likely due to their exposure to human and livestock environments. The detection of resistance genes in MGEs of families previously reported in human streptococci suggests a potential for horizontal gene transfer between animals and humans. These findings underscore the importance of integrated One Health surveillance and antimicrobial stewardship efforts to mitigate the spread of resistance across human, animal, and environmental interfaces.

## Data Availability

Sequence data were deposited in the European Nucleotide Archive under the project accession number PRJEB95797.

## SUPPLEMENTAL MATERIAL

Supplemental material spectrum.02567-25-s0001.pdf Fig. S1 to S4; Tables S1 to S5.

ASM does not own the copyrights to Supplemental Material that may be linked to, or accessed through, an article. The authors have granted ASM a non-exclusive, world-wide license to publish the Supplemental Material files. Please contact the corresponding author directly for reuse.
