# Supplementary material for: Macrolide resistance determinants and their associations in streptococci from selected livestock and wildlife species from Catalonia, Northeast Spain
Source: Microbiol Spectr. 2026 Mar 16;14(4):e02567-25. doi: 10.1128/spectrum.02567-25 (PMC13055226; doi:10.1128/spectrum.02567-25)
Supplement: Supplemental material — Fig. S1 to S4; Tables S1 to S5. [file spectrum.02567-25-s0001.pdf]

Supplementary materials contents

Supplementary Figure S1. Study design diagram 1

Supplementary Figure S2. Phylogenetic tree and presence/absence matrix depicting acquired antibiotic resistance genes associated with resistance of *Streptococcus suis*. 3

Supplementary Figure S3. Clade classification of integrases, relaxases, and VirB4 proteins of Tn*5252*-family ICEs and dICEs 5

Supplementary Figure S4. Schematic representation of prophages carrying resistance genes found in *Streptococcus* spp. from animals 7

Supplementary Table S1. Extended summary of bioinformatic methodology 9

Supplementary Table S2. Metadata of study genomes 11

Supplementary Table S3. Streptococcal species isolated from animals (carriers and infected) from a historical collection of Animal Health Research Center (CReSA-IRTA) and a pet collection from Universitat Autònoma de Barcelona (UAB)......................... 13

Supplementary Table S4. Antimicrobial resistance among macrolide- and/or lincosamide-resistant streptococci 14

Supplementary Table S5. Classification of ICEs and dICEs carrying resistance genes identified in *Streptococcus* spp. from animals 15

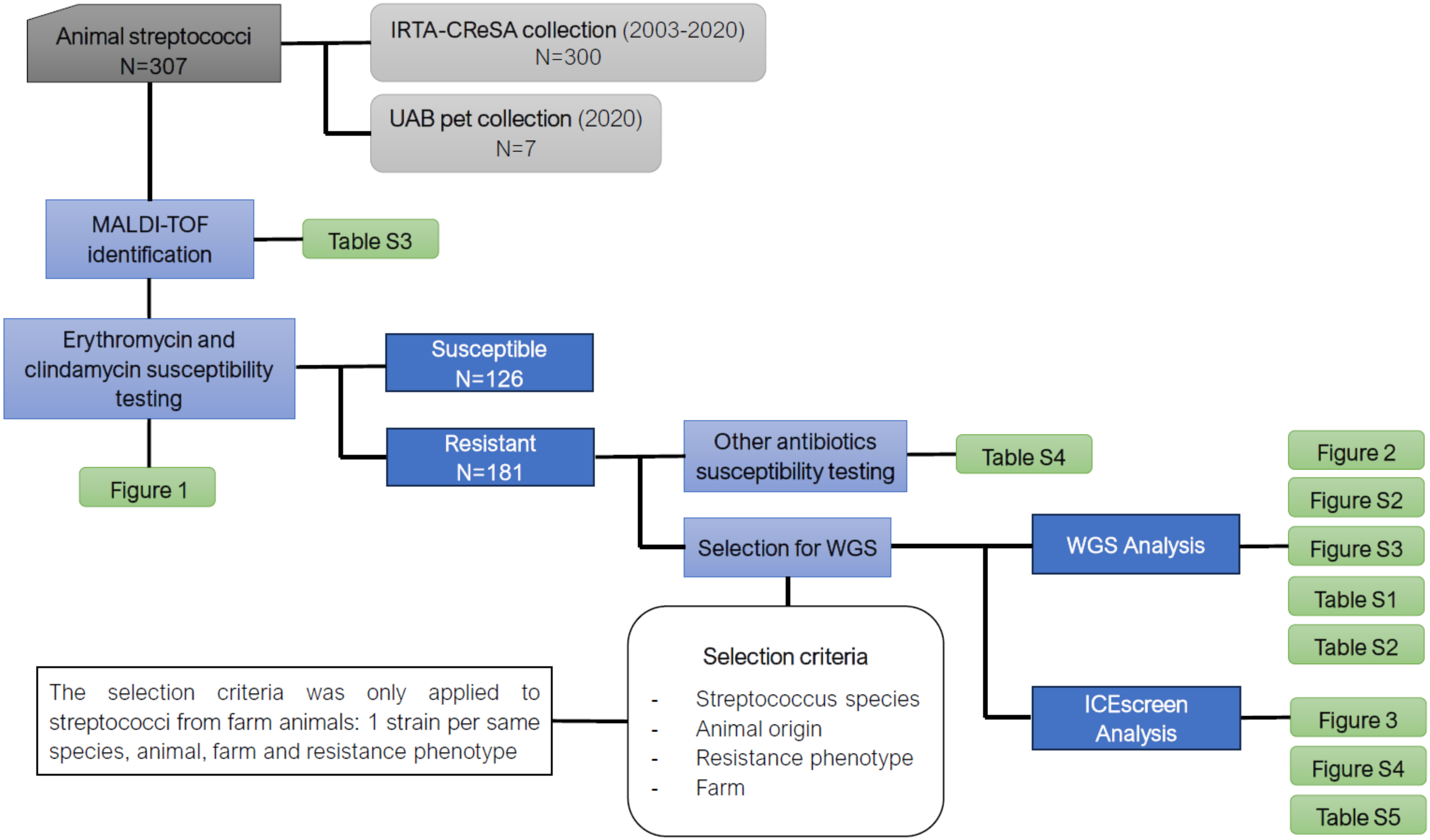
Animal streptococci
N=307
IRTA-CReSA collection (2003-2020)
N=300
UAB pet collection (2020)
N=7
MALDI-TOF identification
Table S3
Erythromycin and clindamycin susceptibility testing
Figure 1
Susceptible
N=126
Resistant
N=181
Other antibiotics susceptibility testing
Table S4
Selection for WGS
WGS Analysis
Figure 2
Figure S2
Figure S3
Table S1
Table S2
ICEscreen Analysis
Figure 3
Figure S4
Table S5
Selection criteria
- Streptococcus species
- Animal origin
- Resistance phenotype
- Farm
The selection criteria was only applied to streptococci from farm animals: 1 strain per same species, animal, farm and resistance phenotype

Supplementary Figure S1. Study design diagram. Schematic representation of flowchart used in this study. Grey represents isolates from the historical collection. Light blue and blue show methodological techniques used in this work and green represents figures and supplementary material. Selection criteria for WGS are also specified in this diagram.

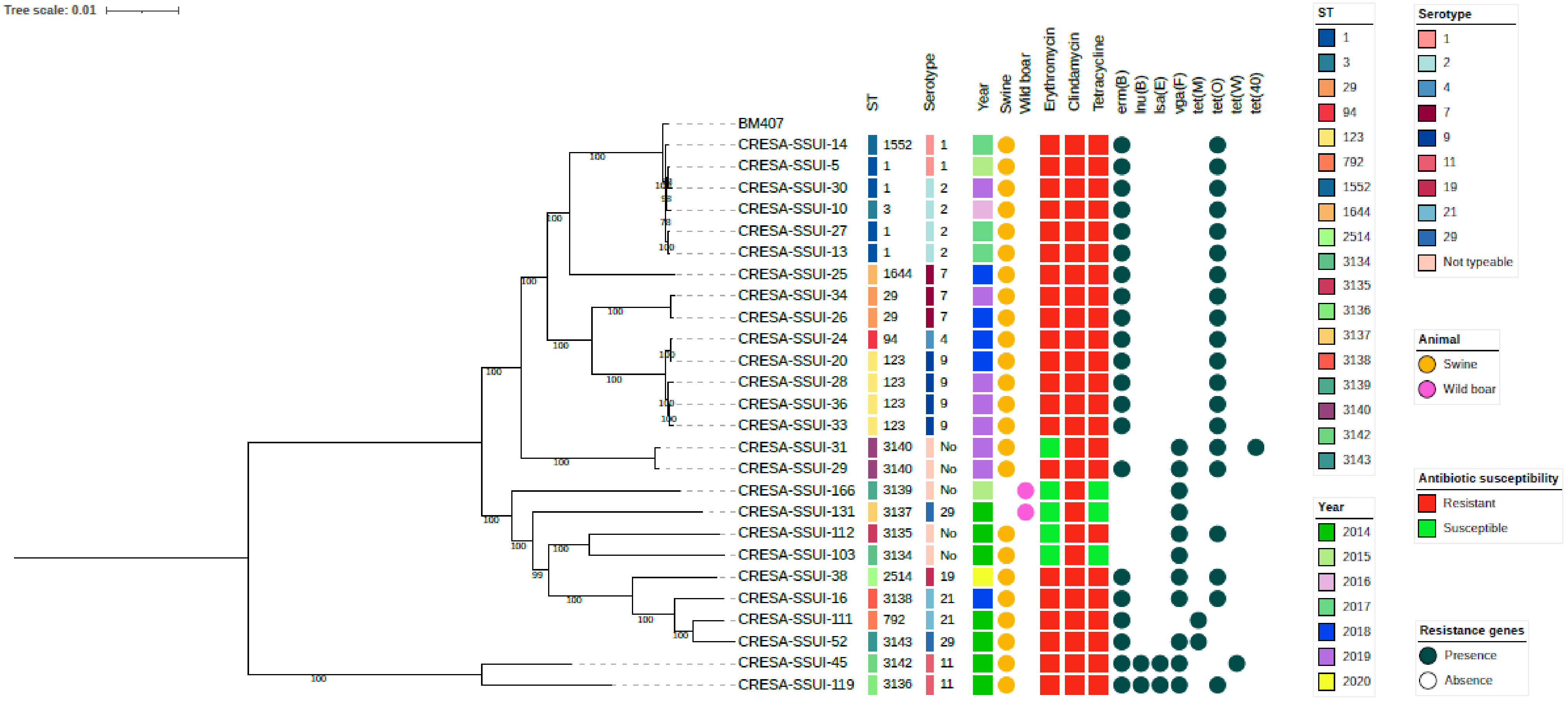

Tree scale: 0.01
BM407
CRESA-SSUI-14
CRESA-SSUI-5
CRESA-SSUI-30
CRESA-SSUI-10
CRESA-SSUI-27
CRESA-SSUI-13
CRESA-SSUI-25
CRESA-SSUI-34
CRESA-SSUI-26
CRESA-SSUI-24
CRESA-SSUI-20
CRESA-SSUI-28
CRESA-SSUI-36
CRESA-SSUI-33
CRESA-SSUI-31
CRESA-SSUI-29
CRESA-SSUI-166
CRESA-SSUI-131
CRESA-SSUI-112
CRESA-SSUI-103
CRESA-SSUI-38
CRESA-SSUI-16
CRESA-SSUI-111
CRESA-SSUI-52
CRESA-SSUI-45
CRESA-SSUI-119
ST
Serotype
Year
Swine
Wild boar
Erythromycin
Clindamycin
Tetracycline
erm(B)
lnu(B)
lsa(E)
vga(F)
tet(M)
tet(O)
tet(W)
tet(40)
Serotype: 1, 2, 4, 7, 9, 11, 19, 21, 29, Not typeable
ST: 1, 3, 29, 94, 123, 792, 1552, 1644, 2514, 3134, 3135, 3136, 3137, 3138, 3139, 3140, 3142, 3143
Animal: Swine, Wild boar
Year: 2014, 2015, 2016, 2017, 2018, 2019, 2020
Antibiotic susceptibility: Resistant, Susceptible
Resistance genes: Presence, Absence

Supplementary Figure S2. Phylogenetic tree and presence/absence matrix depicting acquired antibiotic resistance genes associated with resistance of *Streptococcus suis*. The branches of the phylogenetic tree include the strain identification. The bootstrap values (percentages of 1000 replicates) are depicted at the branching points. The first part includes data of sequence type (ST, coloured rectangles and numbers in the first column), serotype (rectangles of second column), year of isolation (squares of third column), and data of the animal from which streptococci was isolated (coloured circles in the fourth and fifth columns). The second part includes a matrix of columns with squares representing antibiotic susceptibility testing results: red (resistant) and green (susceptible); the antibiotic is referred at the top. The last section shows presence (dark green circle) or absence (no circle) of different acquired resistance genes associated with antibiotic resistance.

Integrases

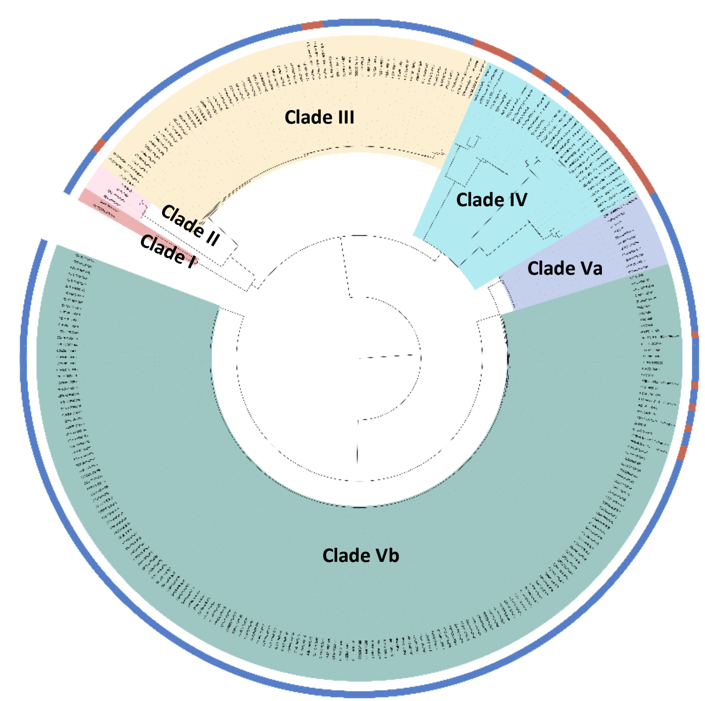

Strain	Element	Integrase clade
CRESA-SCAN-191	Tn*5252*	III
CRESA-SSUI-5	Tn*5252*	III
CRESA-SSUI-10	Tn*5252*	III
CRESA-SSUI-13	Tn*5252*	III
CRESA-SSUI-14	Tn*5252*	III
CRESA-SSUI-16	dICE Tn*5252*	Vb
CRESA-SSUI-20	dICE Tn*5252*	IV
CRESA-SSUI-24	dICE Tn*5252*	IV
CRESA-SSUI-25	Tn*5252*	Vb
CRESA-SSUI-26	dICE Tn*5252*	IV
CRESA-SSUI-27	Tn*5252*	Va
CRESA-SSUI-28	Tn*5252*	IV
CRESA-SSUI-30	Tn*5252*	III
CRESA-SSUI-31	dICE Tn*5252*	IV
CRESA-SSUI-33	Tn*5252*	IV
CRESA-SSUI-34	Tn*5252*	Vb
CRESA-SSUI-36 (1)	Tn*5252*	III
CRESA-SSUI-36 (2)	Tn*5252*	IV
CRESA-SSUI-38	Tn*5252*	III
CRESA-SSUI-45 (2)	dICE Tn*5252*	IV
CRESA-SSUI-112	dICE Tn*5252*	Vb
CRESA-SSUI-119	Tn*5252*	Vb
CRESA-STHO-143	dICE Tn*5252*	Vb

Relaxases

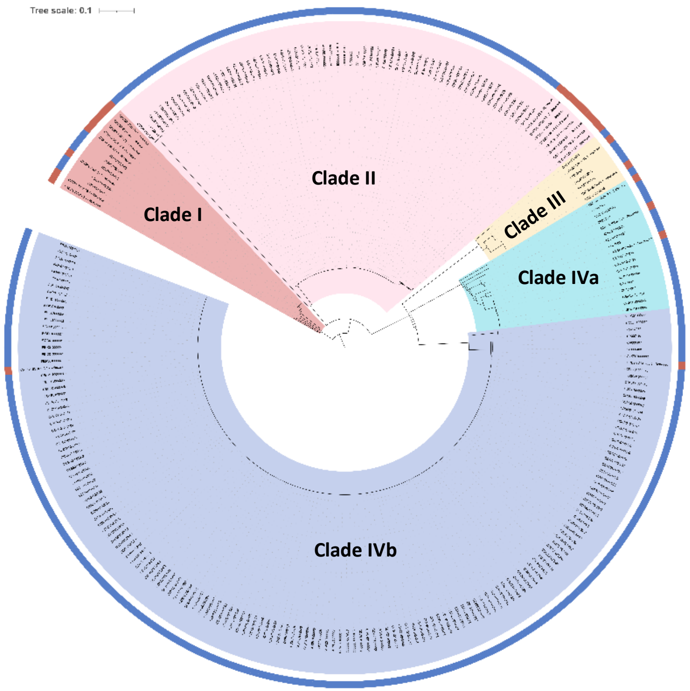

Strain	Element	Relaxase clade
CRESA-SCAN-191	Tn*5252*	II
CRESA-SSUI-5	Tn*5252*	II
CRESA-SSUI-10	Tn*5252*	II
CRESA-SSUI-13	Tn*5252*	II
CRESA-SSUI-14	Tn*5252*	II
CRESA-SSUI-16	dICE Tn*5252*	IVa
CRESA-SSUI-20	dICE Tn*5252*	I
CRESA-SSUI-24	dICE Tn*5252*	I
CRESA-SSUI-25	Tn*5252*	II
CRESA-SSUI-26	dICE Tn*5252*	I
CRESA-SSUI-27	Tn*5252*	IVa
CRESA-SSUI-28	Tn*5252*	I
CRESA-SSUI-30	Tn*5252*	II
CRESA-SSUI-31	dICE Tn*5252*	I
CRESA-SSUI-33	Tn*5252*	I
CRESA-SSUI-34	Tn*5252*	III
CRESA-SSUI-36 (1)	Tn*5252*	IVa
CRESA-SSUI-36 (2)	Tn*5252*	I
CRESA-SSUI-38	Tn*5252*	IVb
CRESA-SSUI-45 (1)	dICE Tn*5252*	I
CRESA-SSUI-112	dICE Tn*5252*	IVb
CRESA-SSUI-119	Tn*5252*	III
CRESA-SUBE-181	dICE Tn*5252*	II

VirB4

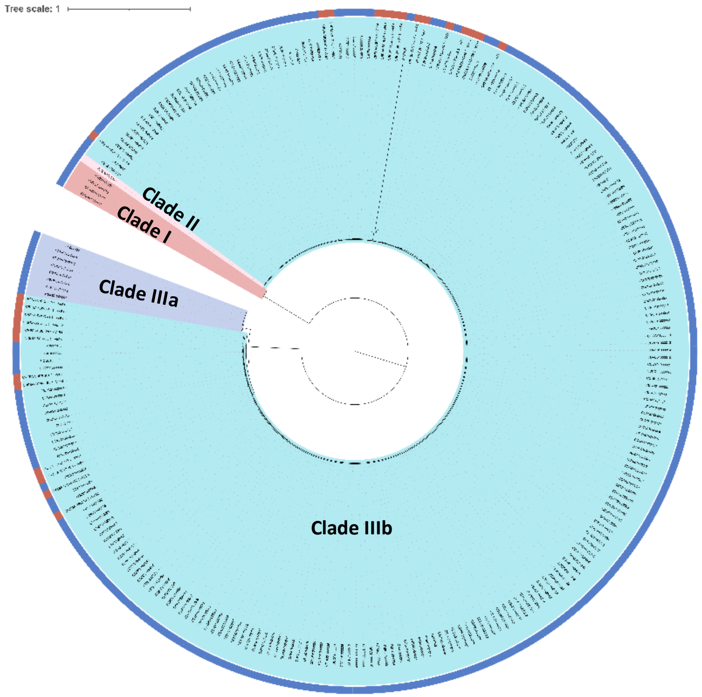

Strain	Element	VirB4 clade
CRESA-SCAN-191	Tn*5252*	IIIb
CRESA-SHYO-41	dICE Tn*5252*	IIIb
CRESA-SHYO-49	dICE Tn*5252*	IIIb
CRESA-SHYO-60	dICE Tn*5252*	IIIb
CRESA-SHYO-139	dICE Tn*5252*	IIIb
CRESA-SHYO-149	dICE Tn*5252*	IIIb
CRESA-SPLU-87	dICE Tn*5252*	IIIb
CRESA-SPLU-127	dICE Tn*5252*	IIIb
CRESA-SSUI-5	Tn*5252*	IIIb
CRESA-SSUI-10	Tn*5252*	IIIb
CRESA-SSUI-13	Tn*5252*	IIIb
CRESA-SSUI-14	Tn*5252*	IIIb
CRESA-SSUI-25	Tn*5252*	IIIb
CRESA-SSUI-27	Tn*5252*	IIIb
CRESA-SSUI-28	Tn*5252*	IIIb
CRESA-SSUI-30	Tn*5252*	IIIb
CRESA-SSUI-33	Tn*5252*	IIIb
CRESA-SSUI-34	Tn*5252*	IIIb
CRESA-SSUI-36 (1)	Tn*5252*	IIIb
CRESA-SSUI-36 (2)	Tn*5252*	IIIb
CRESA-SSUI-38	Tn*5252*	IIIb
CRESA-SSUI-45 (1)	dICE Tn*5252*	IIIb
CRESA-SSUI-119	Tn*5252*	IIIb
CRESA-STHO-143	dICE Tn*5252*	IIIb
CRESA-STRE-121	dICE Tn*5252*	IIIb
CRESA-SUBE-181	dICE Tn*5252*	IIIb

Huang J. et al isolates   Study isolates

Supplementary Figure S3. Clade classification of integrases, relaxases, and VirB4 proteins of Tn*5252*-family ICEs and dICEs. Phylogenetic trees compare integrases, relaxases, and VirB4 proteins from Tn*5252*-family ICEs and dICEs identified in our study with those classified into clades by Huang J. et al (Reference 8 of main manuscript; https://doi.org/10.3389/fcimb.2016.00118.8). Protein sequences were aligned using Clustal Omega and Neighbour-Joining trees were constructed. The coloured outer circle of each tree represents the origin of the isolates: isolates from our study (red) and isolates from Huang J. et al (blue). Below each tree, the classification of integrases, relaxases, and VirB4 proteins belonging to different Tn*5252* ICEs or dICEs strains is shown.

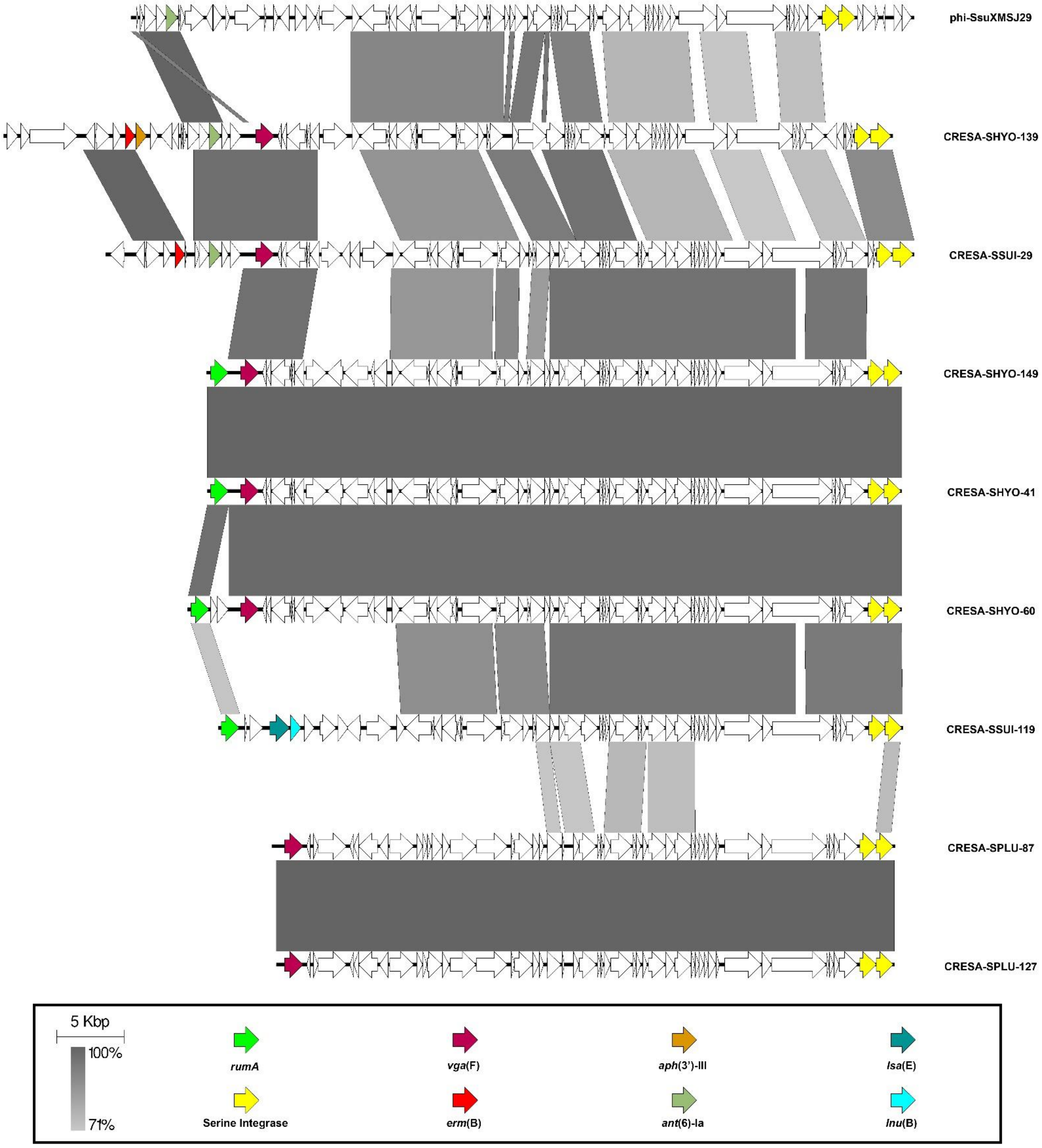
phi-SsuXMSJ29
CRESA-SHYO-139
CRESA-SSUI-29
CRESA-SHYO-149
CRESA-SHYO-41
CRESA-SHYO-60
CRESA-SSUI-119
CRESA-SPLU-87
CRESA-SPLU-127
5 Kbp
100%
71%
rumA
vga(F)
aph(3')-III
lsa(E)
Serine Integrase
erm(B)
ant(6)-Ia
lnu(B)

Supplementary Figure S4. Schematic representation of prophages carrying resistance genes found in *Streptococcus* spp. from animals. This figure shows the prophages containing antibiotic resistance determinants. The prophage phi-SsuXMSJ29 (Acc. No: MZ960489.1) was used as a reference. Grey-to-white shaded areas connect regions based on similarity (identity from 71% to 100%). Arrows depict the genes present in each prophage. Coloured arrows represent different genes: *rumA* (light green), serine integrase (yellow), *vga*(F) (burgundy), *erm*(B) (red), *aph*(3')-III (orange), *ant*(6)-Ia (olive green), *lsa*(E) (dark turquoise) and *lnu*(B) (turquoise).

Supplementary Table S1. Extended summary of bioinformatic methodology

PROGRAM	VERSION	STEP	REFERENCE	PARAMETERS	WEB	PAPER
**BACTOPIA**	3.0.0	Complete analysis of bacterial genomes		Default	https://bactopia.github.io/latest/	1
**BACTOPIA GATHER MODULE**		**Get all the samples into a single place**		Default		
art	2.5.8	For simulating error-free reads for an input assembly		Default	https://www.niehs.nih.gov/research/resources/software/biostatistics/art	2
fastq-scan	1.0.1	Reads a FASTQ from STDIN and outputs summary statistics		Default	https://github.com/rpetit3/fastq-scan	
**BACTOPIA QC MODULE**		**Quality control on Illumina reads**		Default		
fastp	0.23.4	A tool designed to provide fast all-in-one preprocessing for FastQ files		Default	https://github.com/OpenGene/fastp	3
fastqc	0.12.1	A quality control tool for high throughput sequence data		Default	https://www.bioinformatics.babraham.ac.uk/projects/fastqc/	
lighter	1.1.2	A tool for correcting sequencing errors in Illumina reads		Default	https://github.com/mourisl/Lighter	4
**BACTOPIA ASSEMBLER MODULE**		**Assembly of Illumina reads**		Default		
any2fasta	0.4.2	Convert various sequence formats to FASTA		Default	https://github.com/tseemann/any2fasta	
assembly-scan	1.0.0	Summary statistics of an input assembly		Default	https://github.com/rpetit3/assembly-scan	
bwa	0.7.17-r1188	Mapping DNA sequences against a large reference genome		Default	https://github.com/lh3/bwa/	5
flash	1.2.11	Merge paired-end reads		Default	https://ccb.jhu.edu/software/FLASH/	6
megahit	1.2.9	Ultra-fast and memory-efficient NGS assembler		Default	https://github.com/voutcn/megahit	7
samclip	0.4.0	Filter SAM file for soft and hard clipped alignments		Default	https://github.com/tseemann/samclip	
samtools	'1.18'	Interacting with high-throughput sequencing data		Default	https://github.com/samtools/samtools	8
shovill	1.1.0	Assemble bacterial isolate genomes from Illumina paired-end reads		Default	https://github.com/tseemann/shovill	
skesa	2.5.1	Strategic k-mer extension for scrupulous assemblies		Default	https://github.com/ncbi/SKESA	9
spades	3.15.5	Assembly and analysis of sequencing data		Default	https://github.com/ablab/spades	10
velvet	1.2.10	Algorithms for de novo short read assembly		Default	https://github.com/dzerbino/velvet	11
**BACTOPIA SKETCHER MODULE**		**Create a sketch of the contigs, and query databases**		Default		
mash	'2.3'	Fast genome and metagenome distance estimation		Default	https://github.com/marbl/Mash	12
sourmash	4.8.8	Quickly search, compare, and analyze genomic and metagenomic data sets		Default	https://github.com/sourmash-bio/sourmash	13
**BACTOPIA PROKKA**		**Annotate the contigs**		Default		
prokka	1.14.6	Rapid prokaryotic genome annotation		Default	https://github.com/tseemann/prokka	14
**BACTOPIA AMRFINDERPLUS**		**Determine the antibiotic resistance of the contigs and proteins**		Default		
amrfinderplus	3.11.18	Find acquired antimicrobial resistance genes		Default	https://github.com/ncbi/amr	15
amrfinderplus-database	2023-08-08.2			Default		
**BACTOPIA MLST**		**Determine the sequence type of the contigs**		Default		
mlst	2.23.0	Scan contig files against traditional PubMLST typing schemes		Default	https://github.com/tseemann/mlst	
mlst-database	2.23.0-20230907			Default	https://pubmlst.org/	16
**BACTOPIA SNIPPY/ROARY IQTREE MODULE**		**Pangenome/Phylogenetic Analysis**		Default		
snippy	4.6.0	Rapid haploid variant calling and core genome alignment	FM252032.1	Default	https://github.com/tseemann/snippy	
roary	3.13.0	Speed stand alone pan genome pipeline		-s -i 80	https://sanger-pathogens.github.io/Roary/	17
snp-dists	0.8.2	Convert a FASTA alignment to SNP distance matrix		Default	https://github.com/tseemann/snp-dists	
iqtree	2.2.2.7	Efficient and versatile phylogenomic software by maximum likelihood		GTR + F + G4 model<br>1000 UFBoot replicates	https://github.com/Cibiv/IQ-TREE	18
ICEscreen	1.3.2	Conjugative or mobilizable genetic elements annotation		Default	https://icescreen.migale.inrae.fr/	19
Unicycler	v0.4.8	Unicycler is an assembly pipeline for bacterial genomes		Default	https://github.com/rrwick/Unicycler	20
Phaster	online	Phage search tool		Default	https://phaster.ca/	21
iTol	online	Tool for the management, display, annotation and manipulation of phylogenetic and other trees			https://itol.embl.de/	22
Geneious	R9	Molecular biology and sequence analysis tools		Identity >80%<br>Coverage >90%	https://www.geneious.com/updates/geneious-prime-r9-1	

Supplementary Table S1 References

1. Petit III RA, Read TD Bactopia - a flexible pipeline for complete analysis of bacterial genomes. mSystems 5 (2020)
2. Huang W, Li L, Myers JR, Marth GT ART: a next-generation sequencing read simulator. Bioinformatics 28, 593–594 (2012)
3. Chen S, Zhou Y, Chen Y, and Gu J fastp: an ultra-fast all-in-one FASTQ preprocessor. Bioinformatics, 34(17), i884–i890. (2018)
4. Song L, Florea L, Langmead B Lighter: Fast and Memory-efficient Sequencing Error Correction without Counting. Genome Biol. 15(11):509 (2014)
5. Li H Aligning sequence reads, clone sequences and assembly contigs with BWA-MEM. arXiv [q-bio.GN] (2013)
6. Magoč T, Salzberg SL FLASH: fast length adjustment of short reads to improve genome assemblies. Bioinformatics 27.21 2957-2963 (2011)
7. Li D, Liu C-M, Luo R, Sadakane K, Lam T-W MEGAHIT: an ultra-fast single-node solution for large and complex metagenomics assembly via succinct de Bruijn graph. Bioinformatics 31.10 1674-1676 (2015)
8. Li H, Handsaker B, Wysoker A, Fennell T, Ruan J, Homer N, Marth G, Abecasis G, Durbin R The Sequence Alignment/Map format and SAMtools. Bioinformatics 25, 2078–2079 (2009)
9. Souvorov A, Agarwala R, Lipman DJ SKESA: strategic k-mer extension for scrupulous assemblies. Genome Biology 19:153 (2018)
10. Bankevich A, Nurk S, Antipov D, Gurevich AA, Dvorkin M, Kulikov AS, Lesin VM, Nikolenko SI, Pham S, Prjibelski AD, Pyshkin AV, Sirotkin AV, Vyahhi N, Tesler G, Alekseyev MA, Pevzner PA SPAdes: a new genome assembly algorithm and its applications to single-cell sequencing. Journal of computational biology 19.5 455-477 (2012)
11. Zerbino DR, Birney E Velvet: algorithms for de novo short read assembly using de Bruijn graphs. Genome research 18.5 821-829 (2008)
12. Ondov BD, Starrett GJ, Sappington A, Kostic A, Koren S, Buck CB, Phillippy AM Mash Screen: high-throughput sequence containment estimation for genome discovery Genome Biol 20, 232 (2019)
13. Brown CT, Irber L sourmash: a library for MinHash sketching of DNA. JOSS 1, 27 (2016)
14. Seemann T Prokka: rapid prokaryotic genome annotation Bioinformatics 30, 2068–2069 (2014)
15. Feldgarden M, Brover V, Haft DH, Prasad AB, Slotta DJ, Tolstoy I, Tyson GH, Zhao S, Hsu C-H, McDermott PF, Tadesse DA, Morales C, Simmons M, Tillman G, Wasilenko J, Folster JP, Klimke W Validating the NCBI AMRFinder Tool and Resistance Gene Database Using Antimicrobial Resistance Genotype-Phenotype Correlations in a Collection of NARMS Isolates. Antimicrob. Agents Chemother. (2019)
16. Jolley KA, Bray JE, Maiden MCJ Open-access bacterial population genomics: BIGSdb software, the PubMLST.org website and their applications. Wellcome Open Res 3, 124 (2018)
17. Andrew J. Page, Carla A. Cummins, Martin Hunt, Vanessa K. Wong, Sandra Reuter, Matthew T. G. Holden, Maria Fookes, Daniel Falush, Jacqueline A. Keane, Julian Parkhill, "Roary: Rapid large-scale prokaryote pan genome analysis". Bioinformatics 31(22):3691-3693 (2015)
18. Nguyen L-T, Schmidt HA, von Haeseler A, Minh BQ IQ-TREE: A fast and effective stochastic algorithm for estimating maximum likelihood phylogenies. Mol. Biol. Evol. 32:268-274 (2015)
19. Julie Lao, Thomas Lacroix, Gérard Guédon, Charles Coluzzi, Sophie Payot, Nathalie Leblond-Bourget, Hélène Chiapello, ICEscreen: a tool to detect Firmicute ICEs and IMEs, isolated or enclosed in composite structures. NAR Genomics and Bioinformatics, Volume 4, Issue 4, December 2022
20. Wick RR, Judd LM, Gorrie CL, Holt KE. Unicycler: resolving bacterial genome assemblies from short and long sequencing reads. PLoS Comput Biol 2017.
21. Arndt, D., Grant, J., Marcu, A., Sajed, T., Pon, A., Liang, Y., Wishart, D.S. (2016) PHASTER: a better, faster version of the PHAST phage search
22. Letunic I, Bork P. Interactive Tree of Life (iTOL) v6: recent updates to the phylogenetic tree display and annotation tool. Nucleic Acids Res.

Supplementary Table S2. Metadata of study genomes

ID	Accession Short Reads	Accession Long Reads	Contigs	Genome size	N50	rMLST_Specie	ST	*allele 1*	*allele 2*	*allele 3*	*allele 4*	*allele 5*	*allele 6*	*allele 7*	*Suis Serotype*	Year	Animal	Source
CRESA-SAGA-189	ERR15397851	-	37	2080915	4	*S. agalactiae*	23	adhP(5)	pheS(4)	atr(6)	glnA(3)	sdhA(2)	glcK(1)	tkt(3)	-	2014	Dolphin	Central nervous system
CRESA-SALA-190	ERR15397852	ERR16338702	139	1763764	14	*S. alactolyticus*	NA	-	-	-	-	-	-	-	-	2016	Sparrow	Liver+Heart
CRESA-SCAN-191	ERR15397853	-	34	1997467	6	*S. canis*	14	gki(4)	gtr(2)	murI(4)	mutS(4)	recP(6)	xpt(3)	yqiZ(4)	-	2017	Dog	-
CRESA-SENT-194	ERR15397854	ERR16338703	63	1925842	10	*S. entericus*	NA	-	-	-	-	-	-	-	-	2017	Sheep	Nasal swab
CRESA-SHYO-130	ERR15397855	ERR16338704	59	2049502	8	*S. hyovaginalis*	NA	-	-	-	-	-	-	-	-	2014	Wild boar	Nasal swab
CRESA-SHYO-133	ERR15397856	ERR16338705	59	2034596	8	*S. hyovaginalis*	NA	-	-	-	-	-	-	-	-	2014	Swine	Nasal swab
CRESA-SHYO-139	ERR15397857	ERR16338706	53	2017062	9	*S. hyovaginalis*	NA	-	-	-	-	-	-	-	-	2014	Swine	Nasal swab
CRESA-SHYO-149	ERR15397858	ERR16338707	108	2039183	8	*S. hyovaginalis*	NA	-	-	-	-	-	-	-	-	2014	Swine	Nasal swab
CRESA-SHYO-41	ERR15397859	ERR16338708	44	1925436	8	*S. hyovaginalis*	NA	-	-	-	-	-	-	-	-	2014	Swine	Nasal swab
CRESA-SHYO-49	ERR15397860	ERR16338709	63	1947712	9	*S. hyovaginalis*	NA	-	-	-	-	-	-	-	-	2014	Swine	Nasal swab
CRESA-SHYO-60	ERR15397861	ERR16338710	47	1962910	10	*S. hyovaginalis*	NA	-	-	-	-	-	-	-	-	2014	Swine	Nasal swab
CRESA-SHYO-96	ERR15397862	ERR16338711	54	1990205	9	*S. hyovaginalis*	NA	-	-	-	-	-	-	-	-	2014	Swine	Nasal swab
CRESA-STRE-121	ERR15397893	ERR16338736	107	1879266	12	*Streptococcus* spp	NA	-	-	-	-	-	-	-	-	2014	Swine	Nasal swab
CRESA-SPLU-127	ERR15397863	ERR16338712	43	2060939	7	*S. pluranimalium*	NA	-	-	-	-	-	-	-	-	2014	Wild boar	Nasal swab
CRESA-SPLU-87	ERR15397864	ERR16338713	40	2189743	8	*S. pluranimalium*	NA	-	-	-	-	-	-	-	-	2014	Swine	Nasal swab
CRESA-SPOR-161	ERR15397865	ERR16338714	21	1819465	4	*S. porcorum*	NA	-	-	-	-	-	-	-	-	2015	Wild boar	Tonsilla swab
CRESA-SSUI-10	ERR15397866	ERR16338715	52	2041102	7	*S. suis*	3	aroA(1)	cpn60(1)	dpr(1)	gki(16)	mutS(1)	recA(1)	thrA(1)	2	2016	Swine	Cerebroespinal fluid
CRESA-SSUI-103	ERR15397867	ERR16338716	140	2309917	24	*S. suis*	3134	aroA(114)	cpn60(296)	dpr(58)	gki(188)	mutS(82)	recA(182)	thrA(8)	No	2014	Swine	Nasal swab
CRESA-SSUI-111	ERR15397868	ERR16338717	102	2295102	16	*S. suis*	792	aroA(62)	cpn60(45)	dpr(39)	gki(49)	mutS(6)	recA(42)	thrA(34)	21	2014	Swine	Nasal swab
CRESA-SSUI-112	ERR15397869	ERR16338718	163	2382711	27	*S. suis*	3135	aroA(612)	cpn60(360)	dpr(58)	gki(681)	mutS(247)	recA(227)	thrA(194)	No	2014	Swine	Nasal swab
CRESA-SSUI-119	ERR15397870	ERR16338719	119	2213448	21	*S. suis*	3136	aroA(611)	cpn60(637)	dpr(541)	gki(461)	mutS(462)	recA(395)	thrA(496)	11	2014	Swine	Nasal swab
CRESA-SSUI-13	ERR15397871	-	29	2027780	5	*S. suis*	1	aroA(1)	cpn60(1)	dpr(1)	gki(1)	mutS(1)	recA(1)	thrA(1)	2	2017	Swine	Joint biopsy
CRESA-SSUI-131	ERR15397872	ERR16338720	105	2237457	20	*S. suis*	3137	aroA(141)	cpn60(787)	dpr(542)	gki(682)	mutS(380)	recA(58)	thrA(197)	29	2014	Wild boar	Nasal swab
CRESA-SSUI-14	ERR15397873	-	38	2025647	7	*S. suis*	1552	aroA(368)	cpn60(1)	dpr(1)	gki(1)	mutS(1)	recA(1)	thrA(1)	1	2017	Swine	Abdomen biopsy
CRESA-SSUI-16	ERR15397874	ERR16338721	108	2373697	16	*S. suis*	3138	aroA(13)	cpn60(305)	dpr(540)	gki(16)	mutS(6)	recA(198)	thrA(34)	21	2018	Swine	Bronchoalveolar lavage
CRESA-SSUI-166	ERR15397875	ERR16338722	238	2358145	25	*S. suis*	3139	aroA(610)	cpn60(786)	dpr(543)	gki(126)	mutS(680)	recA(149)	thrA(61)	No	2015	Wild boar	Tonsilla swab
CRESA-SSUI-20	ERR15397876	ERR16338723	55	2123045	10	*S. suis*	123	aroA(17)	cpn60(21)	dpr(5)	gki(45)	mutS(44)	recA(22)	thrA(4)	9	2018	Swine	Cerebroespinal fluid
CRESA-SSUI-24	ERR15397877	ERR16338724	50	2088073	8	*S. suis*	94	aroA(8)	cpn60(21)	dpr(5)	gki(45)	mutS(44)	recA(22)	thrA(4)	4	2018	Swine	Lung biopsy
CRESA-SSUI-25	ERR15397878	-	36	2142969	6	*S. suis*	1644	aroA(6)	cpn60(21)	dpr(24)	gki(4)	mutS(1)	recA(10)	thrA(4)	7	2018	Swine	Pericardic biopsy
CRESA-SSUI-26	ERR15397879	ERR16338725	92	2043083	16	*S. suis*	29	aroA(8)	cpn60(30)	dpr(5)	gki(34)	mutS(30)	recA(3)	thrA(25)	7	2018	Swine	Thoracic biopsy
CRESA-SSUI-27	ERR15397880	-	37	2040529	6	*S. suis*	1	aroA(1)	cpn60(1)	dpr(1)	gki(1)	mutS(1)	recA(1)	thrA(1)	2	2017	Swine	Pericardic biopsy + Sinovial fluid
CRESA-SSUI-28	ERR15397881	ERR16338726	55	2154754	8	*S. suis*	123	aroA(17)	cpn60(21)	dpr(5)	gki(45)	mutS(44)	recA(22)	thrA(4)	9	2019	Swine	Nasal swab
CRESA-SSUI-29	ERR15397882	ERR16338727	280	2623136	47	*S. suis*	3140	aroA(34)	cpn60(14)	dpr(37)	gki(33)	mutS(285)	recA(40)	thrA(21)	No	2019	Swine	Vagina
CRESA-SSUI-30	ERR15397883	ERR16338728	39	2025314	5	*S. suis*	1	aroA(1)	cpn60(1)	dpr(1)	gki(1)	mutS(1)	recA(1)	thrA(1)	2	2019	Swine	Joint biopsy + Meningeal biopsy
CRESA-SSUI-31	ERR15397884	ERR16338729	274	2543783	48	*S. suis*	3140	aroA(34)	cpn60(14)	dpr(37)	gki(33)	mutS(285)	recA(40)	thrA(21)	No	2019	Swine	Vagina
CRESA-SSUI-33	ERR15397885	ERR16338730	50	2066141	9	*S. suis*	123	aroA(17)	cpn60(21)	dpr(5)	gki(45)	mutS(44)	recA(22)	thrA(4)	9	2019	Swine	Cerebroespinal fluid
CRESA-SSUI-34	ERR15397886	ERR16338731	350	3913563	51	*S. suis*	29	aroA(8)	cpn60(30)	dpr(5)	gki(34)	mutS(30)	recA(3)	thrA(25)	7	2019	Swine	Joint biopsy
CRESA-SSUI-36	ERR15397887	ERR16338732	62	2146957	9	*S. suis*	123	aroA(17)	cpn60(21)	dpr(5)	gki(45)	mutS(44)	recA(22)	thrA(4)	9	2019	Swine	Cerebral biopsy
CRESA-SSUI-38	ERR15397888	-	120	2258439	19	*S. suis*	2514	aroA(523)	cpn60(85)	dpr(72)	gki(108)	mutS(45)	recA(42)	thrA(62)	19	2020	Swine	Blood
CRESA-SSUI-45	ERR15397889	ERR16338733	133	2236262	15	*S. suis*	3142	aroA(613)	cpn60(637)	dpr(517)	gki(461)	mutS(679)	recA(505)	thrA(495)	11	2014	Swine	Nasal swab
CRESA-SSUI-5	ERR15397890	-	28	2024452	5	*S. suis*	1	aroA(1)	cpn60(1)	dpr(1)	gki(1)	mutS(1)	recA(1)	thrA(1)	1	2015	Swine	Joint fluid
CRESA-SSUI-52	ERR15397891	ERR16338734	105	2309644	16	*S. suis*	3143	aroA(62)	cpn60(45)	dpr(88)	gki(680)	mutS(6)	recA(42)	thrA(34)	29	2014	Swine	Nasal swab
CRESA-STHO-143	ERR15397892	ERR16338735	243	2564898	12	*S. thoraltensis*	NA	-	-	-	-	-	-	-	-	2014	Swine	Nasal swab
CRESA-SUBE-181	ERR15397894	-	61	2067011	3	*S. uberis*	-	arcC(2)	ddl(1)	gki(6)	recP(2)	tdk(~73)	tpi(2)	yqiL(3)	-	2010	Cow	Mastitis
UAB-SEQU-7-1	ERR15397895	-	11	1875680	1	*S. equinus*	NA	-	-	-	-	-	-	-	-	2020	Griffon vulture	Swab
UAB-STRE-10-1	ERR15397896	-	48	1943531	6	*Streptococcus* spp	NA	-	-	-	-	-	-	-	-	2020	Dog	Swab
UAB-STRE-1-3	ERR15397897	-	42	1907231	5	*Streptococcus* spp	NA	-	-	-	-	-	-	-	-	2020	Dog	Swab
UAB-STRE-15-1	ERR15397898	-	93	1877314	18	*Streptococcus* spp	NA	-	-	-	-	-	-	-	-	2020	Dog	Swab
UAB-STRE-4-1	ERR15397899	-	36	2135730	4	*Streptococcus* spp	NA	-	-	-	-	-	-	-	-	2020	Dog	Swab
UAB-STRE-6-2	ERR15397900	-	164	2237323	16	*Streptococcus* spp	NA	-	-	-	-	-	-	-	-	2020	Cat	Swab

Supplementary Table S2. Metadata of study genomes

	MIC (mg/L)				Macrolides				Lincosamides						Tetracyclines					Aminoglycosides					Chloramphenicol
ID	ERY	CLI	TET	CHL	*erm(B)*	*mef(A)*	*mef(E)*	*msr(D)*	*lnu(A)*	*lnu(B)*	*lnu(C)*	*lsa(E)*	*vga(F)*	*lsa(C)*	*tet(M)*	*tet(O)*	*tet(W)*	*tet(40)*	*tet(L)*	*aac(6')*	*aadE*	*ant(6)-Ia*	*aph(2")-IIIa*	*aph(3')-IIIa*	*cat(A)*
CRESA-SAGA-189	>4	<0.5	>4	<4	-	-	+	+	-	-	-	-	-	-	+	-	-	-	-	-	-	-	-	-	-
CRESA-SALA-190	>4	>2	>4	<4	+	-	-	-	-	-	+	-	-	-	+	-	-	-	-	-	-	-	-	-	-
CRESA-SCAN-191	>4	>2	>4	<4	+	-	-	-	-	-	-	-	-	-	-	+	-	-	-	-	+	-	-	-	-
CRESA-SENT-194	>4	>2	>4	<4	+	-	-	-	-	-	-	-	-	-	-	+	-	-	-	-	-	+	-	-	-
CRESA-SHYO-130	>4	>2	>4	<4	+	-	-	-	-	+	-	+	-	-	-	+	-	-	+	-	-	+	-	+	-
CRESA-SHYO-133	>4	>2	>4	<4	+	-	-	-	-	-	-	-	+	-	-	+	-	-	-	-	-	+	+	+	-
CRESA-SHYO-139	>4	>2	>4	<4	+	-	-	-	-	-	-	-	+	-	-	-	+	-	-	-	-	+	-	+	-
CRESA-SHYO-149	>4	>2	>4	>8	+	-	-	-	-	-	-	-	+	-	-	-	+	-	-	-	-	+	-	+	+
CRESA-SHYO-41	>4	>2	>4	>8	+	-	-	-	-	-	-	-	+	-	-	-	+	-	-	-	-	+	-	+	+
CRESA-SHYO-49	>4	>2	>4	<4	+	-	-	-	+	+	-	+	-	-	-	-	+	-	-	-	-	+	-	-	-
CRESA-SHYO-60	>4	>2	>4	>8	+	-	-	-	-	-	-	-	+	-	-	-	+	-	-	-	-	+	-	+	+
CRESA-SHYO-96	>4	>2	>4	<4	+	-	-	-	-	+	-	+	-	-	-	+	-	-	+	-	-	+	-	+	-
CRESA-STRE-121	>4	>2	>4	>8	+	-	-	-	-	+	-	+	-	-	-	+	-	-	-	-	-	+	-	-	+
CRESA-SPLU-127	>4	>2	>4	<4	+	-	-	-	-	+	-	+	+	-	-	-	+	-	-	-	-	+	-	-	-
CRESA-SPLU-87	>4	>2	>4	<4	+	-	-	-	-	+	-	+	+	-	-	-	+	+	-	-	-	+	+	-	-
CRESA-SPOR-161	<0.25	>2	<1	<4	-	-	-	-	-	-	-	-	+	-	-	-	-	-	-	-	-	-	-	-	-
CRESA-SSUI-10	>4	>2	>4	<4	+	-	-	-	-	-	-	-	-	-	-	+	-	-	-	-	-	-	-	-	-
CRESA-SSUI-103	<0.25	>2	<1	<4	-	-	-	-	-	-	-	-	+	-	-	-	-	-	-	-	-	-	-	-	-
CRESA-SSUI-111	>4	>2	>4	<4	+	-	-	-	-	-	-	-	-	-	+	-	-	-	-	-	-	-	-	-	-
CRESA-SSUI-112	<0.25	>2	>4	<4	-	-	-	-	-	-	-	-	+	-	-	+	-	-	-	-	-	-	-	-	-
CRESA-SSUI-119	>4	>2	>4	<4	+	-	-	-	-	+	-	+	+	-	-	+	-	-	-	-	-	-	-	-	-
CRESA-SSUI-13	>4	>2	>4	<4	+	-	-	-	-	-	-	-	-	-	-	+	-	-	-	-	-	-	-	-	-
CRESA-SSUI-131	<0.25	>2	<1	<4	-	-	-	-	-	-	-	-	+	-	-	-	-	-	-	-	-	-	-	-	-
CRESA-SSUI-14	>4	>2	>4	<4	+	-	-	-	-	-	-	-	-	-	-	+	-	-	-	-	-	-	-	-	-
CRESA-SSUI-16	>4	>2	>4	<4	+	-	-	-	-	-	-	-	+	-	-	+	-	-	-	-	-	-	-	-	-
CRESA-SSUI-166	<0.25	>2	<1	<4	-	-	-	-	-	-	-	-	+	-	-	-	-	-	-	-	-	-	-	-	-
CRESA-SSUI-20	>4	>2	>4	<4	+	-	-	-	-	-	-	-	-	-	-	+	-	-	-	-	-	-	-	-	-
CRESA-SSUI-24	>4	>2	>4	<4	+	-	-	-	-	-	-	-	-	-	-	+	-	-	-	-	-	-	-	-	-
CRESA-SSUI-25	>4	<0.5	>4	<4	+	-	-	-	-	-	-	-	-	-	-	+	-	-	-	-	-	+	-	+	-
CRESA-SSUI-26	>4	>2	>4	<4	+	-	-	-	-	-	-	-	-	-	-	+	-	-	-	-	-	-	-	-	-
CRESA-SSUI-27	>4	>2	>4	<4	+	-	-	-	-	-	-	-	-	-	-	+	-	-	-	-	-	-	-	-	-
CRESA-SSUI-28	>4	>2	>4	<4	+	-	-	-	-	-	-	-	-	-	-	+	-	-	-	-	-	-	-	-	-
CRESA-SSUI-29	>4	>2	>4	<4	+	-	-	-	-	-	-	-	+	-	-	+	-	-	-	-	-	+	-	+	-
CRESA-SSUI-30	>4	>2	>4	<4	+	-	-	-	-	-	-	-	-	-	-	+	-	-	-	-	-	-	-	-	-
CRESA-SSUI-31	<0.25	>2	>4	<4	-	-	-	-	-	-	-	-	+	-	-	+	-	+	-	-	-	-	-	-	-
CRESA-SSUI-33	>4	>2	>4	<4	+	-	-	-	-	-	-	-	-	-	-	+	-	-	-	-	-	-	-	-	-
CRESA-SSUI-34	>4	>2	>4	<4	+	-	-	-	-	-	-	-	-	-	-	+	-	-	-	-	-	-	-	-	-
CRESA-SSUI-36	>4	>2	>4	<4	+	-	-	-	-	-	-	-	-	-	-	+	-	-	-	-	-	-	-	-	-
CRESA-SSUI-38	>4	>2	>4	<4	+	-	-	-	-	-	-	-	+	-	-	+	-	-	-	-	-	-	-	-	-
CRESA-SSUI-45	>4	>2	>4	<4	+	-	-	-	-	+	-	+	+	-	-	-	+	-	-	-	-	+	-	+	-
CRESA-SSUI-5	>4	>2	>4	<4	+	-	-	-	-	-	-	-	-	-	-	+	-	-	-	-	-	-	-	-	-
CRESA-SSUI-52	>4	>2	>4	<4	+	-	-	-	-	-	-	-	+	-	+	-	-	-	-	-	-	-	-	-	-
CRESA-STHO-143	>4	>2	>4	<4	+	-	-	-	-	-	-	-	+	-	-	+	-	-	-	-	-	+	+	+	-
CRESA-SUBE-181	>4	>2	>4	<4	+	-	-	-	-	-	+	-	-	-	-	+	-	-	-	-	+	-	-	-	-
UAB-SEQU-7-1	<0.25	<0.5	<1	<4	-	-	-	-	-	-	-	-	-	-	-	-	-	-	-	-	-	-	-	-	-
UAB-STRE-10-1	>4	>2	<1	<4	-	+	-	+	-	+	-	+	-	-	-	-	-	-	-	-	-	-	-	-	-
UAB-STRE-1-3	>4	<0.5	<1	<4	-	+	-	+	-	-	-	-	-	-	-	-	-	-	-	-	-	-	-	-	-
UAB-STRE-15-1	>4	<0.5	<1	<4	-	+	-	+	-	-	-	-	-	-	-	-	-	-	-	-	-	-	-	-	-
UAB-STRE-4-1	>4	>2	<1	<4	-	+	-	+	-	+	-	+	-	-	-	-	-	-	-	-	-	-	-	-	-
UAB-STRE-6-2	>4	>2	<1	<4	+	-	-	-	-	-	-	-	-	+	-	-	-	-	-	+	-	-	-	-	-

Supplementary Table S3. Streptococcal species isolated from animals (carriers and infected) from a historical collection of Animal Health Research Center (CReSA-IRTA) and a pet collection from Universitat Autònoma de Barcelona (UAB)

***Streptococcus* spp**	**Animal**	**N**	**N Total**
S. suis	Swine	112	167
	Wild boar	53	
	Chamois	2	
S. hyovaginalis	Swine	37	43
	Wild boar	3	
	Sheep	3	
S. lutetiensis	Chamois	17	17
Streptococcus spp	Chamois	5	12
	Dog	4	
	Cat	2	
	Swine	1	
S. uberis	Cow	10	10
S. entericus	Sheep	10	10
S. pluranimalium	Swine	4	6
	Wild boar	1	
	Sheep	1	
S. dysgalactiae	Cow	6	6
S. pasteuri	Chamois	5	5
S. gallolyticus	Chamois	5	5
S. agalactiae	Dolphin	2	3
	Dog	1	
S. equinus	Chamois	2	3
	Griffon vulture	1	
S. mitis	Rabbit	1	2
	Chamois	1	
S. ruminantium	Sheep	1	2
	Chamois	1	
S. porcorum	Wild boar	2	2
S. canis	Dog	2	2
S. alactolyticus	Bird	1	1
S. didelphis	Chamois	1	1
S. equi	Sheep	1	1
S. porcinus	Sheep	1	1
S. minor	Chamois	1	1
S. parauberis	Chamois	1	1
S. pyogenes	Rabbit	1	1
S. oralis	Chamois	1	1
S. thoraltensis	Swine	1	1
S. ovis	Chamois	1	1
S. henryi	Chamois	1	1
S. bovis	Cow	1	1
Total			307

Supplementary Table S4. Antimicrobial resistance among macrolide- and/or lincosamide-resistant streptococci

	S. suis		*S. hyovaginalis*		*Streptococcus* spp.		*S. uberis*	*S. entericus*	*S. pluranimalium*		Others	
	Swine	Wild boar	Swine	Wild boar	Dog	Cat	Cow	Sheep	Swine	Wild boar	Others	Total
Tetracycline-resistant	97 (90.6%)	0	35 (100%)	3	0	0	4	2	5	1	5	152 (83.9%)
Co-trimoxazole-resistant	60 (56.1%)	0	15 (42.8%)	3	0	0	0	0	3	1	1	83 (45.8%)
Quinolones-resistant	24 (22.4%)	0	13 (37.1%)	3	0	0	0	0	1	1	1	43 (23.7%)
Penicillin-resistant	5 (4.7%)	0	16 (45.7%)	0	0	0	0	0	0	0	0	21 (11.6%)
Chloramphenicol-resistant	0 (0%)	0	11 (31.4%)	0	0	0	0	0	1	0	0	12 (6.6%)
Number of strains	107	12	35	3	4	2	4	2	5	1	6	181

Supplementary Table S5. Classification of ICEs and dICEs carrying resistance genes identified in *Streptococcus* spp. from animals

Strain	Integrase	Relaxase	Insertion	ICE-family	IMEs inserted	Resistance genes
SCAN-191	Serine	MOBP	*rumA*	Tn*5252*	YES	*erm*(B), *tet*(O), *aadE*
SSUI-10	Serine	MOBP	*rumA*	Tn*5252*	YES (MOBV)	*erm*(B)(x2), *tet*(O)
SSUI-13	Serine	MOBP	*rumA*	Tn*5252*	YES (MOBV)	*erm*(B), *tet*(O)
SSUI-14	Serine	MOBP	*rumA*	Tn*5252*	YES (MOBV)	*erm*(B), *tet*(O)
SSUI-5	Serine	MOBP	*rumA*	Tn*5252*	dIME (MOBV)	*erm*(B), *tet*(O)
SSUI-30	Serine	MOBP	*rumA*	Tn*5252*	YES (MOBV)	*erm*(B), *tet*(O)
SSUI-38	Serine	MOBP	*rumA*	Tn*5252*	YES (MOBV)	*erm*(B), *tet*(O)
SSUI-36 (1)	Serine	MOBP	*rumA*	Tn*5252*	YES (MOBV)	*erm*(B), *tet*(O)
SSUI-25	Tyrosine	MOBP	*rplL*	Tn*5252*	YES (MOBV)	*erm*(B), *tet*(O), *ant*(6)-Ia, *aph*(3')-III
SSUI-27	Tyrosine	MOBP	*rplL*	Tn*5252*	YES (MOBV)	*erm*(B), *tet*(O)
SSUI-119	Tyrosine	MOBP	*rplL*	Tn*5252*	YES (MOBV)	*erm*(B), *tet*(O)
SSUI-34	Tyrosine	MOBP	*rplL*	Tn*5252*	NO	*erm*(B), *tet*(O)
SSUI-28	Serine (3x)	MOBP	*mutT*	Tn*5252*	YES (MOBV)	*erm*(B), *tet*(O)
SSUI-33	Serine (3x)	MOBP	*mutT*	Tn*5252*	YES (MOBV)	*erm*(B), *tet*(O)
SSUI-36 (2)	Serine (3x)	MOBP	*mutT*	Tn*5252*	YES (MOBV)	*erm*(B), *tet*(O)
SAGA-189	Tyrosine	MOBT	-	Tn*916*	NO	*tet*(M)
SSUI-52	Tyrosine	MOBT	-	Tn*916*	NO	*tet*(M)
SSUI-111	Tyrosine	MOBT	-	Tn*916*	NO	*tet*(M)
SUBE-181	-	MOBP	*rumA*	dICE Tn*5252*	YES	*erm*(B), *tet*(O), *aadE*
SHYO-41	-	-	*rumA*	dICE Tn*5252*	YES (MOBV)	*erm*(B), *tet*(W), *aph*(3')-III, *cat*(A)
SPLU-87	-	-	*rumA*	dICE Tn*5252*	NO	*erm*(B), *tet*(W), *tet*(40), *ant*(6)-Ia
SHYO-60	-	-	*rumA*	dICE Tn*5252*	YES (MOBV)	*erm*(B), *tet*(W), *aph*(3')-III, *cat*(A)
SPLU-127	-	-	*rumA*	dICE Tn*5252*	YES (MOBV)	*erm*(B), *tet*(W), *ant*(6)-Ia
SHYO-139	-	-	*rumA*	dICE Tn*5252*	YES (MOBV)	*erm*(B), *tet*(W)
STRE-121	-	-	*rumA*	dICE Tn*5252*	YES (MOBV)	*erm*(B), *tet*(O), *lnu*(B)-*lsa*(E), *ant*(6)-Ia, *cat*(A)
SHYO-49	-	-	*rumA*	dICE Tn*5252*	YES (MOBV)	*erm*(B), *tet*(W)
SHYO-149	-	-	*rumA*	dICE Tn*5252*	YES (MOBV)	*erm*(B), *tet*(W), *ant*(6)-Ia, *aph*(3')-III, *cat*(A)
SSUI-45 (1)	-	-	-	dICE Tn*5252*	NO	*erm*(B), *tet*(W), *ant*(6)-Ia, *aph*(3')-III
SSUI-16	Tyrosine	MOBP	*rplL*	dICE Tn*5252*	YES (MOBV)	*tet*(O)
SSUI-112	Tyrosine	MOBP	*rplL*	dICE Tn*5252*	NO	*tet*(O)
STHO-143	Tyrosine	-	*rplL*	dICE Tn*5252*	dIME (MOBV)	*tet*(O), *aph*(2")-III
SSUI-24	Serine (3x)	MOBP	*mutT*	dICE Tn*5252*	YES (MOBV)	*erm*(B), *tet*(O)
SSUI-20	Serine (3x)	MOBP	*mutT*	dICE Tn*5252*	YES (MOBV)	*erm*(B), *tet*(O)
SSUI-26	Serine (3x)	MOBP	*mutT*	dICE Tn*5252*	YES (MOBV)	*erm*(B), *tet*(O)
SSUI-31	Serine (3x)	MOBP	*mutT*	dICE Tn*5252*	YES (MOBV)	*tet*(O), *tet*(40)
SSUI-45 (2)	Serine (3x)	MOBP	*mutT*	dICE Tn*5252*	NO	*erm*(B), *lnu*(B)-*lsa*(E), *ant*(6)-Ia
